# MOFA+: a statistical framework for comprehensive integration of multi-modal single-cell data

**DOI:** 10.1186/s13059-020-02015-1

**Published:** 2020-05-11

**Authors:** Ricard Argelaguet, Damien Arnol, Danila Bredikhin, Yonatan Deloro, Britta Velten, John C. Marioni, Oliver Stegle

**Affiliations:** 1 European Bioinformatics Institute (EMBL-EBI), Hinxton, Cambridgeshire, CB10 1SD UK; 2 European Molecular Biology Laboratory (EMBL), Heidelberg, Germany; 3 Division of Computational Genomics and Systems Genetics, German Cancer Research Center (DKFZ), Heidelberg, Germany; 4 Cancer Research UK Cambridge Institute, University of Cambridge, Cambridge, CB2 0RE UK; 5 Wellcome Sanger Institute, Hinxton, Cambridge, CB10 1SA UK

**Keywords:** Single cell, Multi-omics, Data integration, Factor analysis

## Abstract

Technological advances have enabled the profiling of multiple molecular layers at single-cell resolution, assaying cells from multiple samples or conditions. Consequently, there is a growing need for computational strategies to analyze data from complex experimental designs that include multiple data modalities and multiple groups of samples. We present Multi-Omics Factor Analysis v2 (MOFA+), a statistical framework for the comprehensive and scalable integration of single-cell multi-modal data. MOFA+ reconstructs a low-dimensional representation of the data using computationally efficient variational inference and supports flexible sparsity constraints, allowing to jointly model variation across multiple sample groups and data modalities.

## Background

Single-cell methods have provided unprecedented opportunities to assay cellular heterogeneity. This is particularly important for studying complex biological processes, including the immune system, embryonic development, and cancer [1–4].

Following the establishment of the first scalable methods for single-cell RNA sequencing (scRNA-seq), other molecular layers are increasingly receiving attention, including single-cell assays for DNA methylation [5–9] and chromatin accessibility [10–12]. More recently, technological advances have enabled multiple biological layers to be probed in parallel in the same cells [12, 13], including single-cell genome and transcriptome (G&T-seq) [14], single-cell DNA methylation and transcriptome (scM&T-seq) [15], single-cell chromatin accessibility and transcriptome (sci-CAR) [16], and single-cell nucleosome, transcriptome and methylation (scNMT-seq) [17], among others [18–24]. These experimental techniques provide the basis for studying regulatory dependencies between transcriptomic and (epi)-genetic diversity at the single-cell level.

However, from a computational perspective, the integration of single-cell assays remains challenging owing to high degrees of missing data, inherent assay noise, and the scale of modern single-cell datasets, which can potentially span millions of cells. Previously, we introduced Multi-Omics Factor Analysis (MOFA) [25], a statistical framework that addresses some of these challenges. However, the inference framework of MOFA is not designed to cope with increasingly large-scale datasets. Moreover, while MOFA is already devised to account for multiple data modalities, this previous model makes strong assumptions about the dependencies across cells and in particular it does not account for side information about the structure between cells, e.g., sample groups, such as batch, donors, or experimental conditions. By pooling and contrasting information across studies or experimental conditions, it would be possible to obtain more comprehensive insights into the complexity underlying biological systems [26–29].

Other methods that have recently been proposed for integrating different data modalities include Seurat (v3) and LIGER, two strategies based on dimensionality reduction and manifold alignment [30, 31]. Both methods anchor independent datasets from related populations of cells by leveraging a common feature space (for example matching gene expression and corresponding promoter accessibility). MOFA+, in contrast, is aimed at a different problem and is designed for integrating data modalities via a common sample space (i.e., measurements derived from the same set of cells), where the features may be distinct across data modalities.

## Results

### Model description

In a previous study, we introduced Multi-Omics Factor Analysis (MOFA), a statistical framework for the integrative analysis of multi-omics data from a common set of samples [25]. Building on the Bayesian Group Factor Analysis framework, MOFA infers a low-dimensional representation of the data in terms of a small number of (latent) factors that capture the global sources of variability. Notably, MOFA employs Automatic Relevance Determination (ARD), a hierarchical prior structure that facilitates untangling variation that is shared across multiple modalities from variability that is present in a single modality. In addition, the sparsity assumptions on the weights facilitate the association of molecular features with each factor. Intuitively, MOFA can be viewed as a statistically rigorous generalization of (sparse) principal component analysis (PCA) to multi-omics data.

While the model is applicable to single-cell assays, MOFA and related factor models have critical limitations, including their scalability and the lack of ability to account for side information about the structure between cells. In particular, these models do not provide a principled approach for integrating multiple sample groups and data modalities within the same inference framework.

Here, we propose MOFA+, a model extension addressing these challenges by (i) developing a stochastic variational inference framework amenable to GPU computations, enabling the analysis of datasets with potentially millions of cells and (ii) incorporating priors for flexible, structure regularization, thus enabling joint modelling of multiple groups and data modalities.

**Fig. 1:**
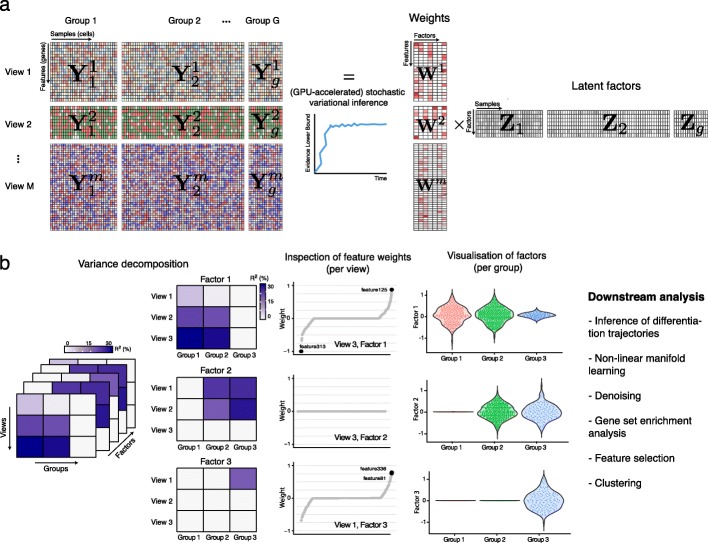
Multi-Omics Factor Analysis v2 (MOFA+) provides an unsupervised framework for the integration of multi-group and multi-view single-cell data. **a** Model overview: the input consists of multiple data sets structured into M views and G groups. Views consist of non-overlapping sets of features that can represent different assays. Analogously, groups consist of non-overlapping sets of samples that can represent different conditions or experiments. Missing values are allowed in the input data. MOFA+ exploits the dependencies between the features to learn a low-dimensional representation of the data (Z) defined by K latent factors that capture the global sources of molecular variability. For each Factor, the weights (W) link the high-dimensional space with the low-dimensional manifold and provide a measure of feature importance. The sparsity-inducing priors on both the factors and the weights enable the model to disentangle variation that is unique to or shared across the different groups and views. Model inference can be significantly sped up using GPU-accelerated stochastic variational inference. **b** The trained MOFA+ model can be queried for a range of downstream analyses: variance decomposition, inspection of feature weights, gene set enrichment analysis, visualization of factors, sample clustering, inference of non-linear differentiation trajectories, denoising and feature selection

Briefly, the inputs to MOFA+ are multiple datasets where features have been aggregated into non-overlapping sets of modalities (also called views) and where cells have been aggregated into non-overlapping sets of groups (Fig. 1a). Data modalities typically correspond to different omics (i.e., RNA expression, DNA methylation, and chromatin accessibility), and groups to different experiments, batches, or conditions. During model training, MOFA+ infers K latent factors with associated feature weight matrices (per data modality) that explain the major axes of variation across the datasets. As in MOFA v1, MOFA+ employs ARD priors to account for structure between views of the data, combined with sparsity-inducing priors to encourage interpretable solutions. However, MOFA+ employs an extended group-wise prior hierarchy, such that the ARD prior does not only act on model weights but also on the factor activities. This strategy enables the simultaneous integration of multiple data modalities and samples groups. Note that if using a single group, the generative model of MOFA+ reduces to the previous MOFA model (but with faster inference). After training, the model output enables a wide range of downstream analyses (Fig. 1b), including variance decomposition, inspection of feature weights, inference of differentiation trajectories, and clustering, among others.

For technical details and mathematical derivations, we refer the reader to “Methods” and the Additional file 2: Supplementary Methods. Guidelines for the selection of group views, data preprocessing and normalization, determination of the number of factors, interpretation of the factor values and the weights are provided in “Methods”. A technical comparison with other factor analysis models is provided in Additional file 3: Table S1.

### Model validation using simulated data

Initially, we validated the new features of MOFA+ using simulated data drawn from its generative model. We considered data representing a range of dataset sizes with differing numbers of data modalities and sample groups.

First, to assess the utility of stochastic variational inference, we trained models either using conventional (deterministic) variational inference (VI) or using stochastic variational inference (SVI). Across a wide range of training hyperparameters (see “Methods”), we observed that SVI yields Evidence Lower Bounds (i.e., the objective function of variational inference) that are consistent with those obtained from conventional variational inference as employed in MOFA (Additional file 1: Fig. S1). However, the GPU-accelerated SVI implementation in MOFA+ achieved up to a ~ 20-fold increase in speed compared to VI, with the most dramatic speedups observed for large datasets. This inference scheme facilitates the application of MOFA+ to datasets comprising hundreds of thousands of cells using commodity hardware (Additional file 1: Fig. S2).

Next, we assessed the group-wise ARD priors, by assessing to what extent it facilitates the identification of factors with simultaneous differential activity between groups and data modalities. Indeed, when simulating data where factors explain differing amounts of variance across groups and across data modalities, MOFA+ was able to more accurately reconstruct the true factor activity patterns than MOFA v1 or conventional Bayesian Factor Analysis (Additional file 1: Fig. S3).

### Integration of a heterogeneous time-course single-cell RNA-seq dataset

To illustrate the ability of MOFA+ to model data with samples that exhibit an explicit group structure, we considered a time-course scRNA-seq dataset, consisting of 16,152 cells that were isolated from multiple mouse embryos at embryonic days E6.5, E7.0, and E7.25 (two biological replicates per stage). In this dataset, individual embryos are expected to exhibit transcriptional differences at different stages and even between embryos from the same stage due to variation in the rate of the developmental progression. As a proof of principle, we used MOFA+ to disentangle stage-specific variation from variation that is shared across all stages. For this purpose, we considered the six batches of cells (two replicates for each of the three embryonic stages) as different groups in the MOFA+ model.

**Fig. 2:**
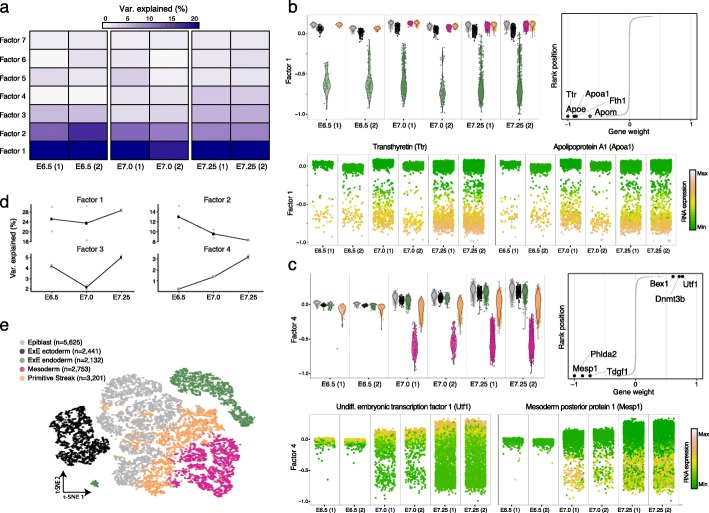
Integration of heterogeneous scRNA-seq experiments reveals stage-specific transcriptomic signatures associated with cell type commitment in mammalian development. **a** The heatmap displays the percentage of variance explained for each Factor (rows) in each group (pool of mouse embryos at a specific developmental stage, columns). **b**, **c** Characterization of Factor 1 as extra-embryonic (ExE) endoderm formation (**b**) and Factor 4 as Mesoderm commitment (**c**). In each panel, the top left plot shows the distribution of Factor values for each batch of embryos. Cells are colored by cell type. Line plots (top right) show the distribution of gene weights, with the top five genes with largest (absolute) weight highlighted. The bottom beeswarm plots represent the distribution of Factor values, with cells colored by the expression of the genes with highest weight. **d** Line plots show the percentage of variance explained (averaged across the two biological replicates) for each Factor as a function of time. The value of each replicate is shown as gray dots. **e** Dimensionality reduction using t-SNE on the inferred factors. Cells are colored by cell type

MOFA+ identified 7 factors that explain at least 1% of variance, which collectively explain between 35 and 55% of the total transcriptional cell-to-cell variance per embryo (Additional file 1: Fig. S4). Some factors recapitulate the existence of post-implantation developmental cell types, including extra-embryonic (ExE) cell types (Factor 1 and Factor 2, respectively) and the transition of epiblast cells to nascent mesoderm via a primitive streak transcriptional state (Factor 4; Fig. 2b, c and Additional file 1: Fig. S5). Consistently, the top weights for these factors are enriched for lineage-specific gene expression markers, including *Ttr* and *Apoa1* for ExE endoderm, *Rhox5* and *Bex3* for ExE ectoderm, and *Mesp1* and *Phlda2* for nascent mesoderm [32]. Other factors captured technical variation due to metabolic stress that affects all batches in a similar fashion (Factor 3, Additional file 1: Fig. S6).

When inspecting the factor activity across developmental stages, we observed that the percentage of variance explained by Factor 1 is not correlated with developmental progression, indicating that commitment to ExE endoderm fate occurs early in the embryo and that the proportion of this cell type remains relatively constant from E6.5 to E7.25. In contrast, the amount of variance explained by Factor 4 increases over time (Fig. 2d), consistent with a higher proportion of cells committing to mesoderm after ingression through the primitive streak.

Altogether, this application shows how MOFA+ can identify biologically relevant structure in scRNA-seq datasets with multiple groups. Interpretability is achieved at the expense of reduced information content per factor (due to the linearity assumption of the model). Nevertheless, the MOFA+ factors can also be used as input for other methods that infer non-linear manifolds that discriminate cell types (Fig. 2e) and enable the reconstruction of pseudotime trajectories [33, 34].

### Identification of context-dependent methylation signatures associated with cellular diversity in the mammalian cortex

As a second use case, we applied MOFA+ to investigate variation in epigenetic signatures between populations of neurons. This use case illustrates how a multi-group and multi-modal structure can be defined from seemingly uni-modal data, which allows for testing specific biological hypotheses.

We analyzed 3069 cells isolated from the frontal cortex of young adult mice, where DNA methylation was profiled using single-cell bisulfite sequencing [7]. Recent studies have demonstrated that neurons contain significant levels of non-CpG methylation (mCH), an epigenetic mark that has been historically dismissed as a methodological artifact of incomplete bisulfite conversion [35–38].

Here we used MOFA+ to dissect the degree of coordination between mCH and mCG signatures in different regions of the brain. As input data we quantified mCH and mCG levels at gene bodies, promoters and putative enhancer elements (“Methods”). Each combination of genomic and sequence context (e.g., mCG at enhancer elements) was defined as a separate data modality. To explore the influence of the neuron’s location, we grouped cells according to their cortical layer: Deep, Middle, or Superficial (Additional file 1: Fig. S7). Low coverage of DNA methylation per cell results in large amounts of missing values, which hampers the use of conventional dimensionality reduction techniques such as PCA or NMF [33, 34, 39]. By contrast, the probabilistic framework underlying MOFA+ naturally accounts for missing values [25].

**Fig. 3:**
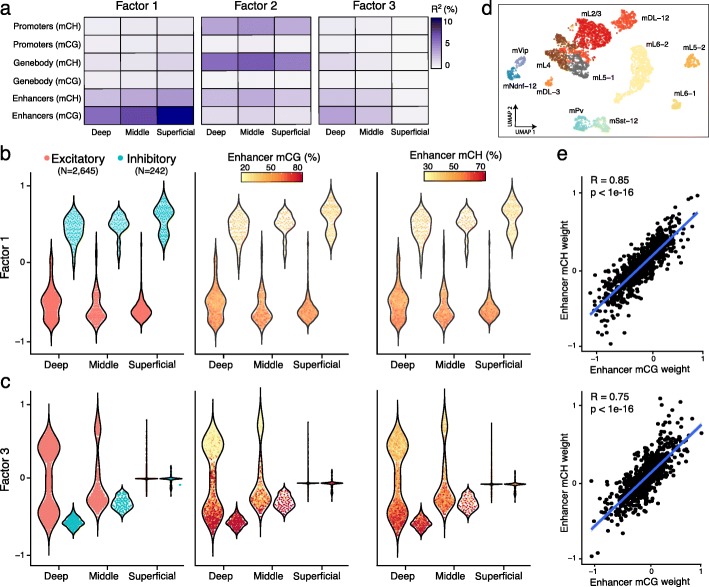
MOFA+ reveals context-dependent DNA methylation signatures associated with cellular diversity in the mammalian cortex. **a** Percentage of variance explained for each Factor across the different groups (cortical layer, *x*-axis) and views (genomic context, *y*-axis). For simplicity, only the first three factors are shown. **b**, **c** Characterization of (**b**) Factor 1 as the two major neuron populations and (**c**) Factor 3 as increased cellular diversity of excitatory neurons in deep cortical layers. The beeswarm plots show the distribution of Factor values for each group, defined as the neuron’s cortical layer. In the left plot, cells are colored by neuron class. In the middle and right plots, the cells are colored by average mCG and mCH levels (%), respectively, of the top 100 enhancers with the largest weights. **d** UMAP projection of the MOFA factors. Each dot represents a cell, colored by maximally resolved cell type assignments. **e** Correlation of enhancer mCG weights (*x*-axis) and mCH weights (*y*-axis) for Factor 1 (top) and Factor 3 (bottom)

MOFA+ identified 5 factors with a minimum variance explained of 1% (Methods; Additional file 1: Fig. S8). Factor 1, the major source of variation, is linked to the division between inhibitory and excitatory neurons. This factor shows significant mCG activity across all cortical layers, primarily associated with coordinated changes in enhancer elements, but to some extent also gene bodies (Fig. 3a,b). Consistently, the top weights in mCG gene body are enriched for genes whose RNA expression has been shown to discriminate between the two classes of neurons, including *Neurod6* and *Nrgn* [7]. In addition, this analysis identified novel genes with differential gene body mCG levels that may have yet unknown roles in defining the epigenetic landscape of neuronal diversity, including *Vsig2*, *Taar3*, and *Cort* (Additional file 1: Fig. S9).

Factor 2 captures genome-wide differences in global mCH levels (*R* = 0.99), which is moderately correlated with differences in global mCG levels (*R* = 0.32) (Additional file 1: Fig. S10). Factor 3 captures heterogeneity linked to the increased cellular diversity along cortical depth, with the Deep layer displaying significantly more diversity of excitatory cell types than the Superficial layer (Fig. 3a,c). Again, we observed that the MOFA+ factors can be used as input to infer non-linear manifolds and reveal the existence of subpopulations of both excitatory and inhibitory cell types (Fig. 3d). Notably, t-SNE representation inferred using MOFA+ factors were substantially better at discriminating subpopulations than the conventional approach of using principal component analysis (Additional file 1: Fig. S11).

Interestingly, in addition to the dominant mCG signal, MOFA+ connected Factor 1 and Factor 3 to variation in mCH, which suggests a putative role of mCH in cellular diversity. We hypothesize that this can be supported if the genomic regions that show mCH signatures are different from the ones marked by the conventional mCG signatures. To investigate this, we correlated the mCH and mCG feature weights for each factor and genomic context. In all cases, we observe a strong positive dependency (Fig. 3e and Additional file 1: Fig. S12), indicating that mCH and mCG signatures are spatially correlated and target similar loci.

Taken together, our results support the hypothesis that mCH and mCG tag the same genomic loci and are associated with the same sources of variation, suggesting that the presence of mCH may be the result of non-specific de novo methylation as a by-product of the establishment of mCG [35].

### MOFA+ reveals molecular signatures of lineage commitment during mammalian embryogenesis

As a final use case, we applied MOFA to a complex dataset with multiple sample groups and modalities. Briefly, scNMT-seq was used to jointly assay RNA expression, DNA methylation, and chromatin accessibility in 1828 cells collected across three stages of mouse development [40]. MOFA+ provides a principled approach for delineating coordinated variation between the transcriptome and the epigenome, and for assigning specific covariance patterns to developmental stages.

As input to the model, we quantified DNA methylation and chromatin accessibility at two sets of regulatory elements: gene promoters and enhancer elements (defined as distal H3K27ac sites [40–42]). RNA expression was quantified for protein-coding genes. After data processing (“Methods”), separate data modalities were defined for the RNA expression and for each combination of genomic context and epigenetic readout (five data modalities in total). Sample groups were defined by considering cells across the developmental stages (E5.5, E6.5, and E7.5), reflecting the underlying experimental design (Additional file 1: Fig. S13). Notably, the epigenetic readouts are extremely sparse, with, on average, only 18% and 10% of cells having recorded data at a gene promoter for DNA methylation and chromatin accessibility, respectively. In this context, methods that pool information across cells and features are essential for robust inference.

**Fig. 4:**
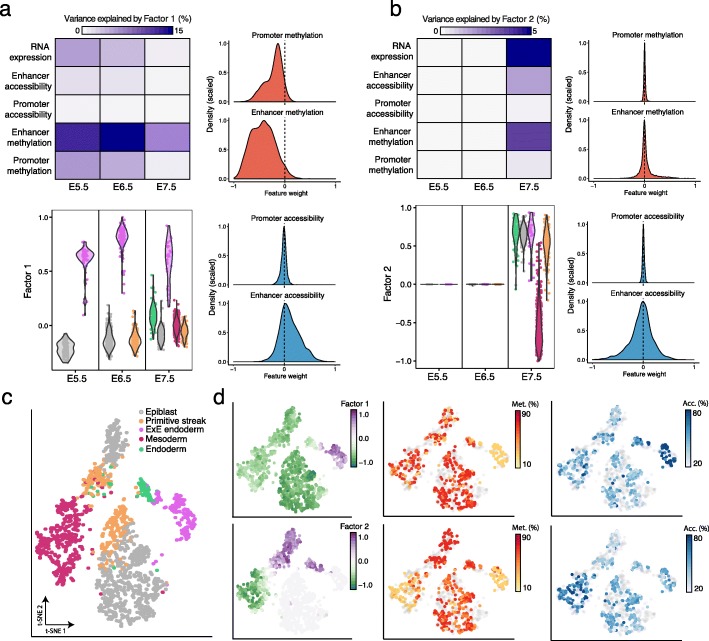
MOFA+ integrates a multi-modal mouse gastrulation atlas to reveal epigenetic signatures associated with lineage commitment. **a**, **b** Characterization of Factor 1 as ExE endoderm formation and Factor 2 as Mesoderm commitment. Top left plot shows the percentage of variance explained by the Factor across the different views (rows) and groups (embryonic stages, as columns). Bottom left plot shows the distribution of Factor values for each stage, colored by cell type assignment. Histograms display the distribution of DNA methylation and chromatin accessibility weights for promoters and enhancer elements. **c** Dimensionality reduction using t-SNE on the inferred MOFA factors. Cells are colored by cell type. **d** Same as (**c**), but cells are colored by Factor 1 values (top left) and Factor 2 values (bottom left); by the DNA methylation levels of the enhancers with the largest weight in Factor 1 (top middle) and Factor 2 (bottom middle); by the chromatin accessibility levels of the enhancers with the largest weight in Factor 1 (top right) and Factor 2 (bottom right)

MOFA+ identified 10 factors that explain at least 1% of variation in gene expression (Additional file 1: Fig. S14). Factor 1 captures the formation of ExE endoderm, a cell type that is present across all stages (Fig. 4a), in agreement with our previous results using the independently generated transcriptomic atlas of mouse gastrulation (Fig. 2). MOFA+ links Factor 1 to changes across all molecular layers. Notably, the distribution of weights for DNA methylation is skewed towards negative values (at both enhancers and promoters), indicating that ExE endoderm cells are characterized by a state of global demethylation, consistent with previous studies [43].

The following factors captured the molecular variation associated with the emergence of the primary germ layers at E7.5: mesoderm (Factor 2, Fig. 4b), and embryonic endoderm (Factor 4, Additional file 1: Fig. S15). Again, for both factors, MOFA+ connected the transcriptome variation to changes in DNA methylation and chromatin accessibility. Yet, in striking contrast to Factor 1, the variance decomposition analysis and the distribution of weights indicate that the epigenetic dynamics are primarily associated with enhancer elements. In contrast, little coordinated variation is observed in promoters (Fig. 4b), even for genes that show strong differential expression between germ layers (Additional file 1: Fig. S16). These results are in agreement with other studies that have identified distal regulatory elements as a major target of epigenetic modifications during embryogenesis [44–46].

The remaining factors capture variation that is mostly driven by the RNA expression, whose etiology can be related to the existence of morphogenic gradients (Factor 8, Additional file 1: Fig. S17), the emergence of other cellular subpopulations during gastrulation (Factor 7, Additional file 1: Fig. S18) and cell cycle (Factor 6, Additional file 1: Fig. S19).

In conclusion, the MOFA+ output suggests that independent cell fate commitment events undergo different modes of epigenetic variation. While some lineages manifest global changes in the epigenetic landscape (ExE endoderm, Factor 1), other cell types are associated with the emergence of local epigenetic patterns that are driven by specific genomic contexts (embryonic endoderm and mesoderm, Factors 2 and 4).

## Discussion

As single-cell technologies mature, they are applied to generate data sets with increasingly complex experimental designs [16, 17, 24, 47, 48]. Consequently, there is a need for integrative computational frameworks that can robustly and systematically interrogate the data generated in order to reveal the underlying sources of variation [26].

In this study, we introduced MOFA+, a generalization of the MOFA framework [25] that facilitates analysis of large-scale datasets with complex multi-group and/or multi-modal experimental designs. From a technical perspective, MOFA+ provides two major features: first, GPU-accelerated stochastic variational inference ensures scalability to potentially millions of cells; second, the use of sparsity priors and hierarchical variance regularization provides a principled approach to analyze data sets that are structured into multiple data modalities and/or multiple groups of samples. Additionally, MOFA+ inherits all the features from its predecessor, including a natural approach for handling missing values as well as the capacity to perform inference with non-Gaussian readouts [25].

Although MOFA+ represents an important step forward in the analysis of single-cell omics data, it also has limitations. First, it requires multi-modal measurements from the same set of cells. This contrasts with other integrative frameworks such as Seurat [31] or LIGER [30], which anchor data sets based on the assumption of a common feature space (e.g., matching gene expression and promoter accessibility). Second, the model is only able to capture moderate non-linear relationships (Additional file 1: Fig. S20). We speculate that this could be addressed by combining MOFA+ with concepts from variational autoencoders, as recently proposed for the analysis of scRNA-seq data [49–51]. Third, the model currently assumes independence between features in its prior distributions, despite the fact that genomic features are known to interact via complex regulatory networks [52].

## Conclusions

In this study, we introduced MOFA+, a statistical framework aimed at the large-scale datasets with complex experimental designs that include multiple groups of features (i.e., data modalities) and multiple groups of cells (i.e., sample groups). We applied MOFA+ to single-cell data sets of different scales and designs. To facilitate adoption of the method, we deploy MOFA+ as open-source software with multiple tutorials and a web-based analysis workbench, enabling a user-friendly in-depth characterization of multi-modal single-cell data.

## Methods

### Multi-Omics Factor Analysis v2 model (MOFA+)

The input to MOFA+ is a list of matrices, each matrix corresponding to specific group and data modality (see Fig. 1 for a visual representation).

We introduce the following notation: *M* for the number of data modalities, *D*_*m*_ for the number of features in the *m*th modality, *G* for the number of sample groups, *N*_*g*_ for the number of samples in the *g*th group, and *K* for the number of factors.

As in the original version of MOFA [25], the underlying master equation is the standard matrix factorization framework:

$$ {\mathrm{Y}}_{\mathrm{g}\mathrm{m}}={\mathrm{Z}}_{\mathrm{g}}{\mathrm{W}}_{\mathrm{m}}^T+{\epsilon}_{\mathrm{g}\mathrm{m}} $$

- *Y*_*gm*_ denotes the matrix of observations for the *m*th modality and the *g*th group.
- *W*_*m*_ denotes the weight matrix for the *m*th modality
- *Z*_*g*_ denotes the factor matrix for the *g*th group
- *ε*_*gm*_ denotes the residual noise for the *m*th modality and the *g*th group. The specific form of the noise can be tailored to the nature of the data type [25]

The factor matrix *Z*_*g*_ has dimensionality (*N*_*g*_,*K*) and contains the low-dimensional representation of the samples from the *g*th group. The weight matrix *W*_*m*_ has dimensionality (*D*_*m*_,*K*) and contains an association score for each feature with each factor. The noise matrix *ε*_*gm*_ contains the unexplained variance (i.e., noise) for each feature in each group.

The model is formulated in a probabilistic Bayesian setting. We introduce prior distributions on all unobserved variables of the model in order to induce specific regularization criteria, as described below in the section “Model regularization”.

### Interpretation of the factors

The MOFA+ factors capture the global sources of variability in the data. Mathematically, each factor ordinates cells along a one-dimensional axis centered at zero. Samples with different signs manifest opposite phenotypes along the inferred axis of variation, with higher absolute value indicating a stronger effect. Note that the interpretation of factors is analogous to the interpretation of the principal components in PCA.

### Interpretation of the weights

The weight matrices provide a score for how strong each feature relates to each factor, hence allowing a biological interpretation of the MOFA+ factors. Features with no association with the factor have values close to zero, while genes with strong association with the factor have large absolute values. The sign of the weight indicates the direction of the effect: a positive weight indicates that the feature has higher levels in the cells with positive factor values, and vice versa.

### Model regularization

The regularization of the weights and the factors is critical to enable MOFA to perform inference with data sets that consists of multiple data modalities and/or groups of samples. In the original version of MOFA, hierarchical priors were applied to the weights to enable inference and interpretable outputs of multi-modal data sets. In MOFA+, we generalized this by introducing a symmetric regularization for both the factors and weights, hence accounting for structure in both the sample space and the feature space (see Additional file 2: Supplementary Methods for mathematical details).

In more detail, we combine two levels of regularization. The first level consists of an Automatic Relevance Determination (ARD) prior to explicitly model differential activity of factors across data modalities and/or across sample groups. The second level consists of a spike-and-slab prior to simultaneously push individual weights and factor values to zero. The latter encourages sparse solutions where factors are (potentially) associated with a small number of active features and/or active within small subsets of samples.

### Stochastic variational inference

In MOFA, inference was performed using mean-field variational Bayes (VI) [53–55]. While this framework is typically faster than sampling-based Monte Carlo approaches, it becomes prohibitively slow when applied to large single-cell datasets. In MOFA+, we implemented a stochastic version of the algorithm (SVI) [55, 56] that can be accelerated by performing computation using GPUs. Importantly, our implementation of the stochastic algorithm is efficient only when the number of samples (cells) is significantly larger than the number of features. Otherwise, we advise the user to perform standard VI.

Mathematically, the use of SVI is based on redefining the coordinate ascent optimization problem in VI in terms of a (natural) gradient ascent problem that can be described by the following equation:

$$ {\mathrm{x}}^{\left(t+1\right)}={\mathrm{x}}^{(t)}+{\rho}^{(t)}\nabla F\left({\mathrm{x}}^{(t)}\right) $$

where *x* represents the variables to be inferred and *F*(*x*) is the objective function, in this case the Evidence Lower Bound (ELBO). In the stochastic inference framework, a fast approximation of the gradient is calculated using a random subset of the data (a batch). To ensure a smooth convergence, the step size *ρ*(*t*) is adjusted at each iteration using the following equation:

$$ {\rho}^{(t)}=\frac{\tau }{{\left(1+\kappa t\right)}^{3/4}} $$

where *τ* defines the starting learning rate and *k* controls its rate of decay (forgetting rate). Hence, the use of SVI comes at the cost of introducing additional hyperparameters: a batch size (as a percentage of the full data set), a starting learning rate and a forgetting rate. A trade-off exists where large batch sizes lead to a more precise estimate of the gradient, but they are more computationally expensive to calculate. While we find the hyperparameters to be relatively robust in simulated data (Additional file 1: Fig. S1), we advise the user to do model selection by a grid-search approach. By default, we use GPU-accelerated standard variational inference if the full data set fits into the GPU memory. Otherwise, we perform stochastic variational inference using a batch size of 50%, a starting learning rate of 1.0 and a forgetting rate of 0.25. Convergence is achieved when the difference in the ELBO between iteration *i* and iteration *i − 1* is less than 1e−4.

For a full mathematical derivation of the SVI algorithm, we refer the reader to Additional file 2: Supplementary Methods.

### Variance decomposition

Once the model is trained, the variance explained by each factor *k* in each sample group *g* and in each data modality *m* is calculated using a coefficient of determination:

$$ {R^2}_{gm k}=1-{\left(\sum \limits_{n,d}\left({Y}_{gm}-{W}_m{Z}_g\right)\right)}^2/{\left(\sum \limits_{n,d}{Y}_{gm}\right)}^2 $$

### Non-Gaussian likelihoods

MOFA+ supports a variety of different likelihood models to enable integration of diverse combinations of data types. These include a Gaussian noise model for continuous data, a Poisson model for count data and a Bernoulli model for binary data. This feature is inherited from MOFA [25]. To implement efficient variational inference in conjunction with non-Gaussian likelihoods (Poisson or Bernoulli), we adapt prior work using local variational bounds [57]. This feature is inherited from the first MOFA model, and we refer the reader to [25] for mathematical details. This approach requires the introduction of additional parameters which significantly slows down model training (Additional file 1: Fig. S21). We advise the user to apply data transformations and use a Gaussian likelihood when possible.

### Determining the number of factors

The selection of the number of factors is an important parameter of the training procedure.

In MOFA+, we have implemented Automatic Relevance Determination priors (see Additional file 2. Supplementary Methods) to automatically learn the effective number of factors. Hence, the user only has to specify the starting number of factors, and factors that do not explain any variation will be pruned during model inference. After the model is trained, the user can manually apply a filtering and remove factors that explain less than a pre-specified value of variance (either in each data modality or across all data modalities). This filtering will depend on the data set and the aim of the analysis. To get an overview on the major sources of variability, a small number of factors (*K* < 10) is sufficient. For other purposes, such as imputation, even small sources of variability are important to be captured and the threshold on variance explained should be lowered to retrieve a large number of factors.

### Model selection

The optimization procedure of MOFA+ depends on the parameter initialization and is hence not guaranteed to find the same exact solution at every trial. Hence, when using random initialization, factors can vary between different model instances and a model selection step using the ELBO is advised. However, to simplify model training and interpretation in our implementation, we eliminated the random component by initialising the factors using the principal components from the concatenated data set.

### Guidelines for data processing

Appropriate normalization during the data processing steps is critical for an optimal model fit. The user should normalize the data according to the likelihood model that will be adopted, which will typically be a Gaussian distribution. In this case, for count-based assays such as (single-cell) RNA-seq, we recommend size factor normalization followed by a variance stabilization transformation [58].

We also advise the users to perform a feature selection step by subsetting highly variable features. The aim of this step is to reduce the feature imbalance between different views, simplify the model interpretation and speed up the training procedure.

Finally, undesired technical sources of variation that should not be captured by the MOFA+ factors should be regressed out a priori. Typical examples are mitochondrial content or the number of expressed genes in scRNA-seq data. Alternatively, if the technical variation is driven by batch effects and the user is interested in exploring the heterogeneity between batches, we advise the users to use the batch label as grouping criteria.

### Guidelines for the selection of groups

Groups are typically based on the experimental design (i.e., conditions and batches), but the user can also explore data-driven groups. There is no “right” or “wrong” definition of groups, but some definitions will be more useful than others. Importantly, the aim of the multi-group framework is not to capture differential changes in mean levels between the groups (as for example when doing differential RNA expression). The aim is to find out which sources of variability are shared between the different groups and which ones are exclusive to a single group. To achieve this, the features are centered per group (i.e., intercept effects are regressed out) before fitting the model.

It is important to note that the size of the group can influence the reconstruction of factors. In general, the more samples per group, the more complexity there will exist in the dataset, which can manifest itself in retrieval of a higher number of factors.

### Guidelines for the selection of data modalities

Data modalities typically correspond to different molecular layers, but the user can also explore data-driven modalities that do not necessarily correspond to different molecular readouts (see for example Fig. 3). Analogous to the number of samples per group, the size of the data modality can have an influence on the latent space, such that larger data modalities can contribute more to the latent space than small data modalities, simply because they have larger amounts of variation. The signal that can be extracted from small data modalities will depend on the degree of structure within the dataset, the levels of noise and on how strong the sample imbalance is between data modalities. Hence, in the case of a strong feature imbalance, we recommend the user to subset highly variable features in the large data modalities to maintain the number of features within the same order of magnitude.

### Gene set enrichment analysis

Gene set enrichment analysis was performed using the Reactome gene sets [59]. For every gene set G, we evaluate its significance via a parametric *t*-test, where we contrast the weights of the foreground set (features that belong to the set G) versus the background set (the weights of features that do not belong to the set G). Resulting *P* values were adjusted for multiple testing for each factor using the Benjamini–Hochberg procedure [60]. Significant enrichments were at a false discovery rate of 1%.

### Data processing for the scRNA-seq application

Cells were subset to stages E6.5, E7.0, and E7.25. Cells from stage E6.75 were not included in the analysis because they consist of a single biological replicate. Gene expression counts were normalized using *scran* [61], and they were modelled in MOFA with a Gaussian likelihood. A comparison with a Poisson likelihood model is shown in Additional file 1: Fig. S21. The 5000 most overdispersed genes after regressing out the stage effect were selected prior to fit the model. Details on the quality control and data preprocessing can be found in [32].

### Data processing for the single-cell DNA methylation application

DNA methylation was quantified over genomic features using a binomial model where the number of successes is the number of reads that support methylation (or accessibility) and the number of trials is the total number of reads. A CpG methylation rate was calculated for each genomic feature and cell using a maximum likelihood approach. The rates were subsequently transformed to M-values [62] and modelled with a Gaussian likelihood.

As input to MOFA+, we filtered genomic features with low coverage (at least 3 CpG measurements or at least 10 CpH measurements) and we selected the intersection of the top 5000 most variable sites across the different genomic and sequence contexts (see Additional file 1: Fig. S8). Details on the quality control and data preprocessing can be found in [7].

### Data processing for the scNMT-seq application

Gene expression counts were quantified over protein-coding genes using featureCounts [63] with the Ensembl gene annotation 87 [64]. The read counts were log-transformed and size-factor adjusted and modelled with a Gaussian likelihood. As input to MOFA+, we filtered genes with a dropout rate higher 90% and we subsetted the top 5000 most variable genes (after regressing out the stage effect). In addition, batch effects and the dropout rate per cell were regressed out prior to fitting the model.

DNA methylation and chromatin accessibility data were quantified over genomic features using a binomial model where the number of successes is the number of reads that support methylation (or accessibility) and the number of trials is the total number of reads. A CpG methylation or GpC accessibility rate for each genomic feature and cell was calculated by maximum likelihood. The rates were subsequently transformed to M-values [62] and modelled with a Gaussian likelihood. As input to MOFA+, we filtered genomic features with low coverage (at least 3 CpG and 5 GpC measurements) and we selected the top 2500 most variable sites per combination of genomic context and data modality (see Additional file 1: Fig. S14). Details on the quality control and data preprocessing can be found in [40].

## Supplementary information

**Additional file 1.** Supplementary Figures S1-S21.

**Additional file 2.** Supplementary Methods.

**Additional file 3.** Supplementary Table 1, theoretical comparison with previous methods.

**Additional file 4.** Review history.

## Data Availability

MOFA+ is implemented as both Python and R packages, and it is freely available under the LGPL-3.0 license on GitHub (https://github.com/bioFAM/MOFA2) [65]. The specific MOFA+ release used for the results presented in this manuscript is archived on zenodo [66]. The repository includes vignettes and source code to reproduce the analyses presented in this article. The datasets analyzed in this study are available from the Gene Expression Omnibus (GEO) repository under the following accession numbers: GSE87038 [33], GSE97179 [7], and GSE121708 [41].
