# Supplementary material for: MOFA+: a statistical framework for comprehensive integration of multi-modal single-cell data
Source: Genome Biol. 2020 May 11;21:111. doi: 10.1186/s13059-020-02015-1 (PMC7212577; doi:10.1186/s13059-020-02015-1)
Supplement: Supplementary file 1 — Additional file 1. Supplementary Figures S1-S21. [file 13059_2020_2015_MOESM1_ESM.pdf]

Supplementary Figures for the manuscript

MOFA+: a probabilistic framework for comprehensive integration of multi-modal single-cell data

Ricard Argelaguet, Damien Arnol, Danila Bredikhin,
Yonatan Deloro, Britta Velten, John C Marioni, Oliver Stegle

April 1, 2020

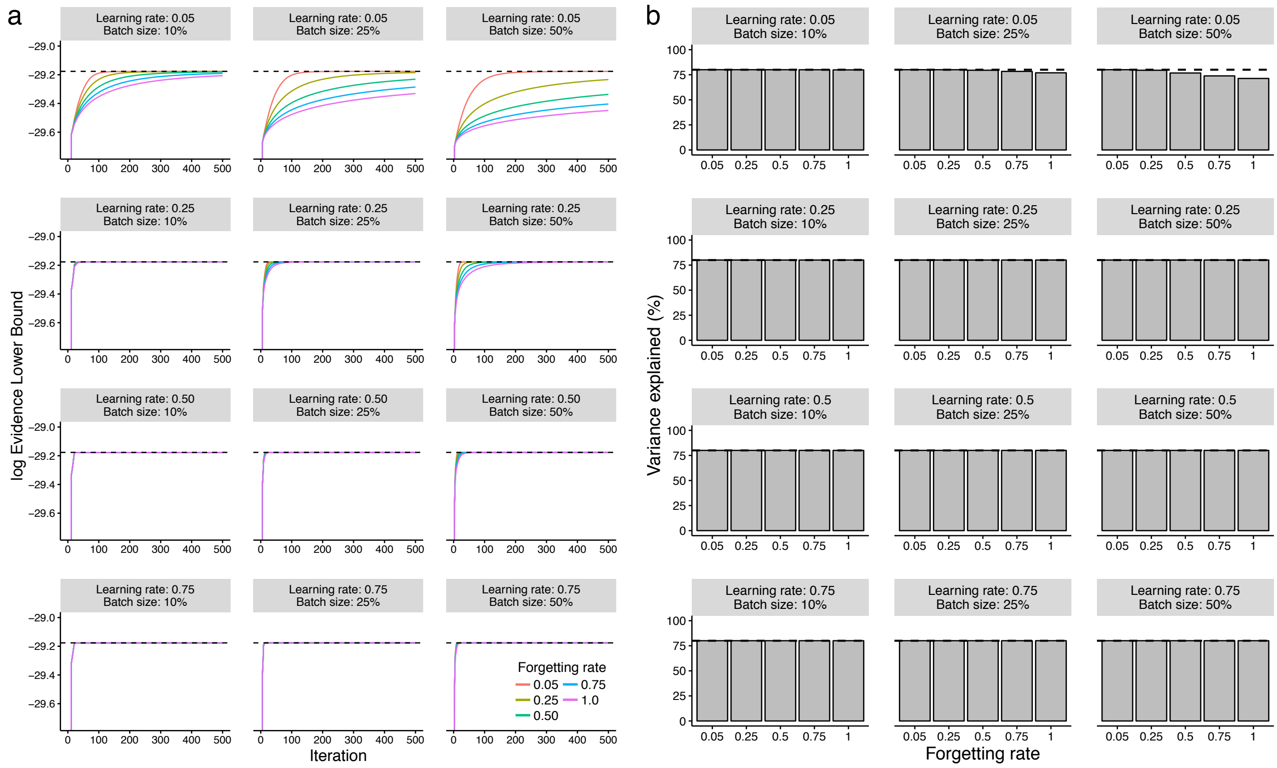

Fig. S1: Validation of stochastic variational inference using simulated data.
Data is simulated from the generative model with the following parameters: $M = 3$ views, $G = 3$ groups, $D = 1,000$ features (per view), $N = 100,000$ samples (per group) and $K = 25$ factors.
(a) Line plots display the iteration number of the inference (x-axis) and the log- Evidence Lower Bound (ELBO) on the y-axis. Panels correspond to different values of batch sizes (10%, 25%, 50% of the data) and initial learning rates (0.05, 0.25, 0.50, 0.75). Colours correspond to different forgetting rates (0.05, 0.25, 0.50, 0.75, 1.0). The dashed horizontal line indicates the ELBO achieved using standard variational inference.
(b) Bar plots display the forgetting rate (x-axis) and the total variance explained (%) in the y-axis. Panels correspond to different values of batch sizes (10%, 25%, 50% of the data) and initial learning rates (0.05, 0.25, 0.50, 0.75). The dashed line indicates the variance explained (%) achieved using standard variational inference.

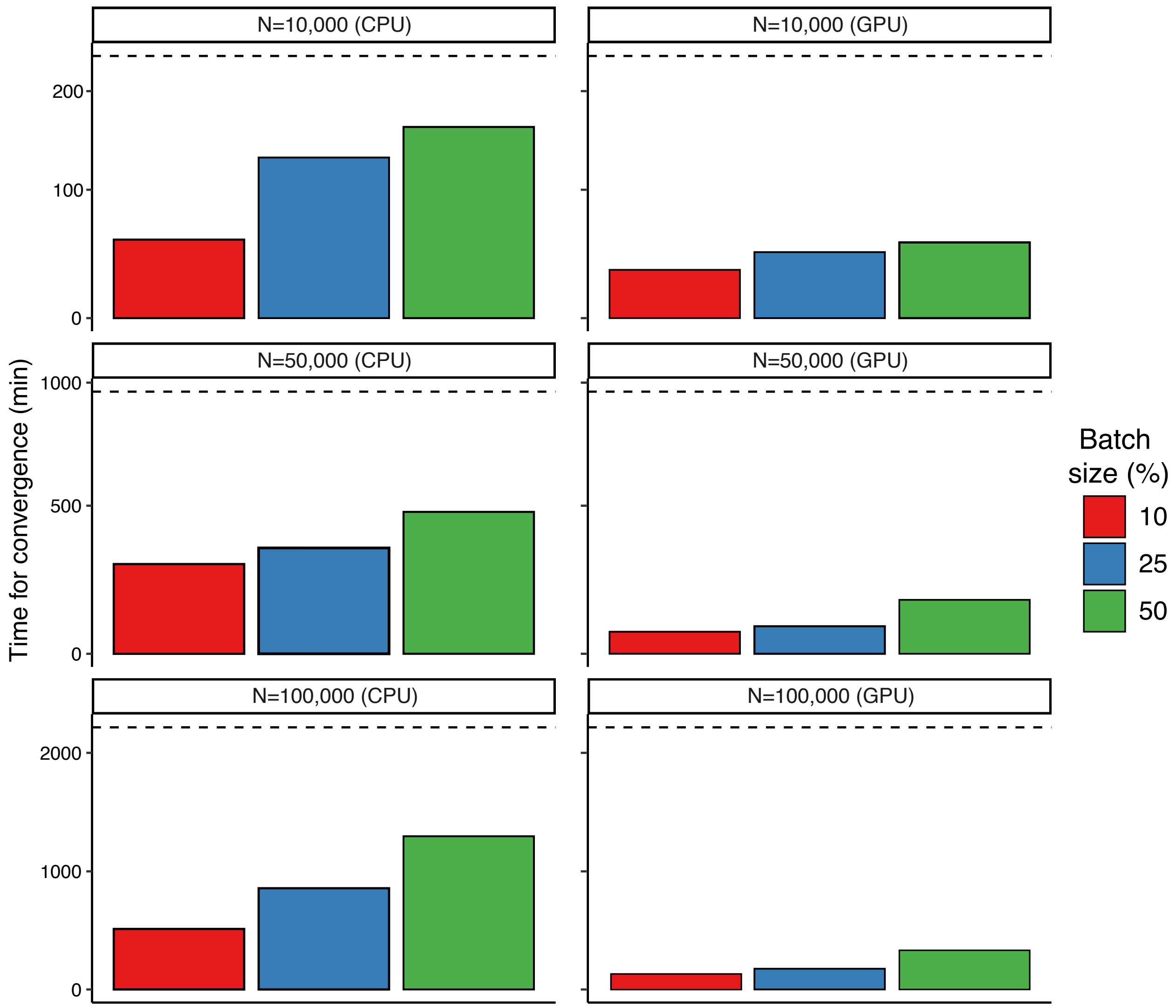

Fig. S2: Evaluation of convergence speed for stochastic variational inference using simulated data.

Data is simulated from the generative model with increasing sample size from $N = 1,000$ to $N = 100,000$. The other parameters are fixed to $M = 3$ views, $G = 3$ groups, $D = 1,000$ features (per view), and $K = 25$ factors. Bar plots show the training time for standard variational inference (VI) and for stochastic variational inference (SV). Colors represent stochastic models trained with different batch sizes (10%, 25% or 50%). Learning rate and forgetting rate hyperparameters were both fixed to 0.5. VI models were fit using a single E5-2680v3 CPU. SVI models were fit using an Nvidia GTX 1080Ti GPU.

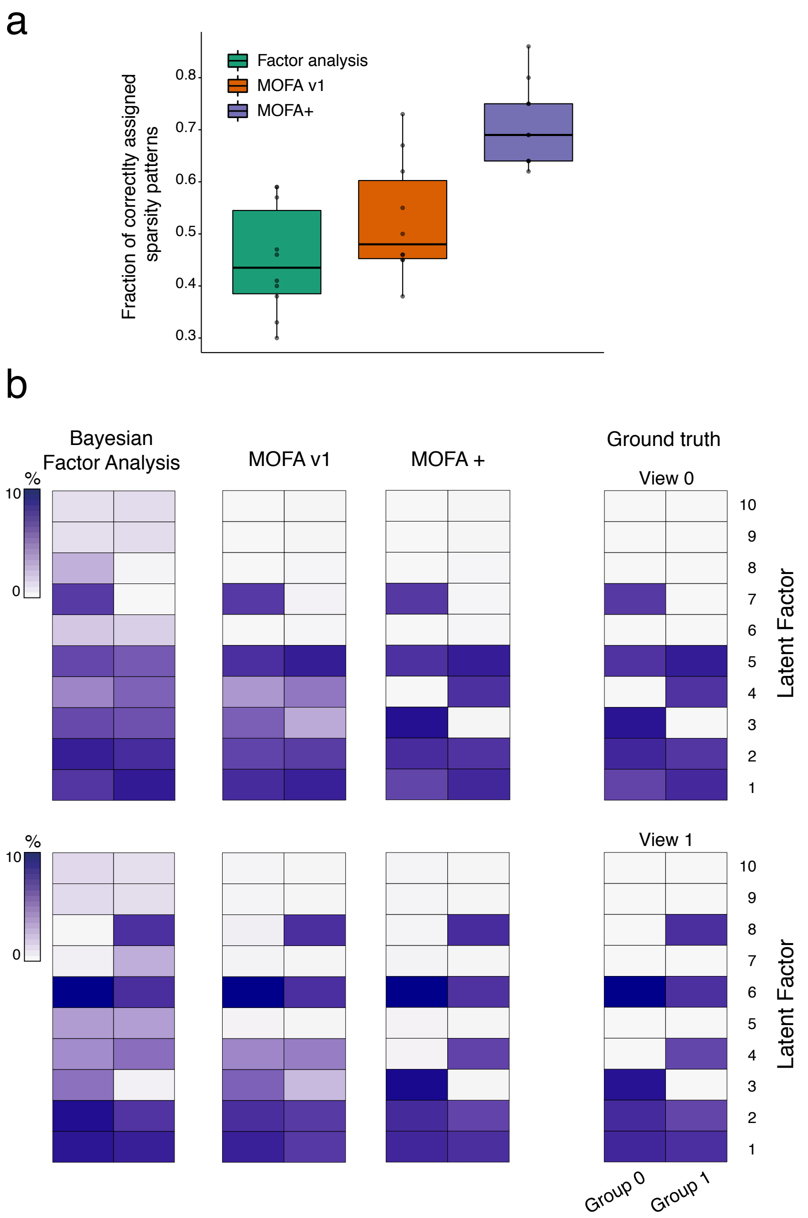

Fig. S3: Validation of group-wise ARD prior in the factors using structured simulated data. Data is simulated from the MOFA+ generative model with the following parameters: M=2 views, G=2 groups, D=1,000 features, N=1,000 samples and K=10 factors. We incorporate structure in the simulation process by turning some factors off in random sets of views and groups. The task of MOFA+ is to recover the true factor activity structure given a random initialisation. We compared three models: Bayesian Factor Analysis (no sparsity priors), MOFA v1 (only view-wise sparsity prior) and MOFA+ (view-wise and group-wise sparsity prior).

(a) Fraction of correctly assigned sparsity patterns for each model. If the difference between the simulated and the inferred variance explained pattern was less than 0.1%, the pattern was assigned as correct. The box plots show median levels and the first and third quartile out of 10 trials. Whiskers show 1.5x the interquartile range.

(b) Representative example of the resulting variance explained patterns. The first row of heatmaps correspond to view 0 and the second row to view 1. In each heatmap, the first column corresponds to group 0 and the second column to group 1. Rows correspond to the inferred factors. The colour scale displays the fraction of variance explained by a given factor in a given view and group. The heatmaps displayed in columns one to three show the solutions yielded by different models (Bayesian Factor Analysis; MOFA; MOFA+). The ground truth is shown in the right panel.

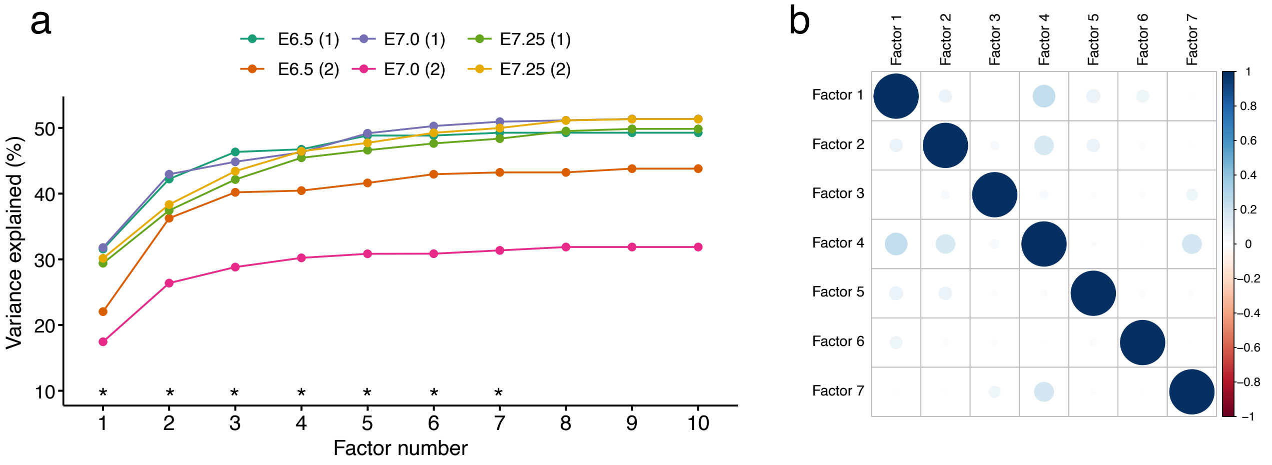

Fig. S4: Unsupervised characterisation of MOFA+ factors from the gastrulation scRNA-seq data set.
(a) Cumulative variance explained (per group, y-axis) versus factor number (x-axis). Asterisks indicate the factors that are selected for downstream analysis (minimum of 1% variance explained). (a) Pearson correlation coefficients between selected factors. In MOFA+ there are no orthogonality constraints, but the factors are expected to be largely uncorrelated.

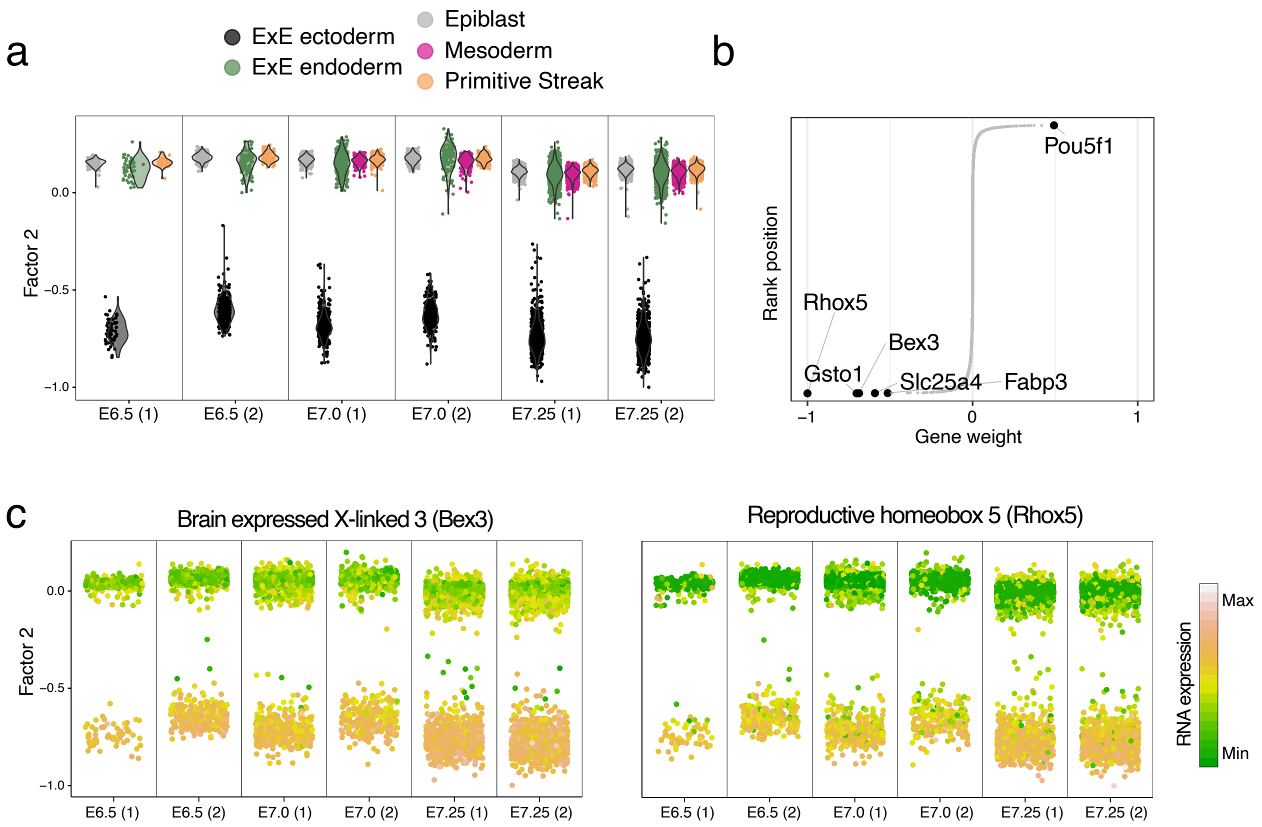

Fig. S5: Characterisation of Factor 2 as extra-embryonic (ExE) ectoderm formation.
(a) Distribution of factor values per batch of embryos, where each dot represents a single cell, coloured by cell type.
(b) Distribution of gene weights, with the top six genes with largest (absolute) weight highlighted.
(c) Distribution of factor values per batch of embryos, with cells coloured by the expression of the genes with highest weight.

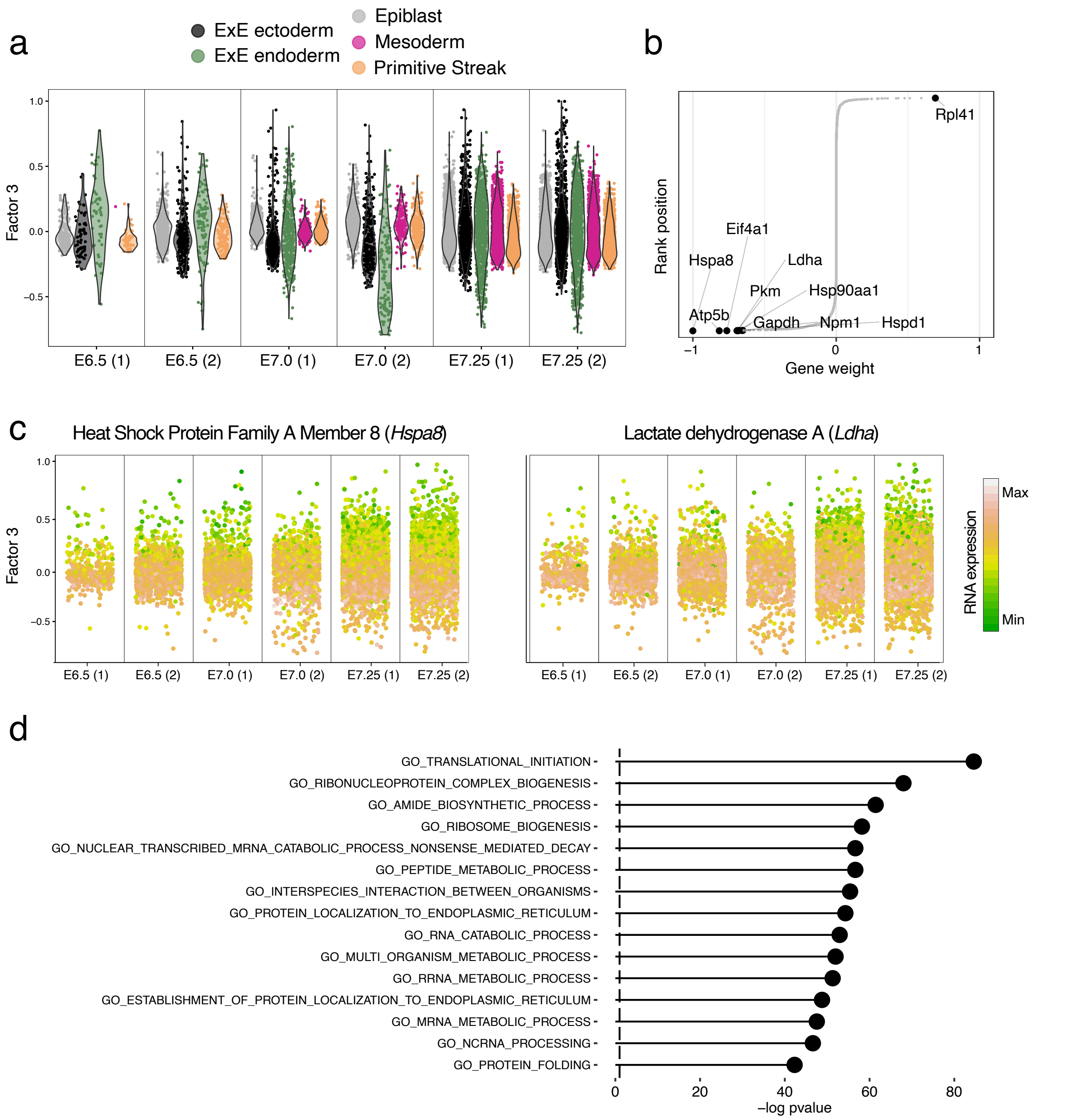

Fig. S6: Characterisation of Factor 3 as cell-to-cell differences in the metabolic response.
(a) Distribution of factor values per batch of embryos, coloured by cell type.
(b) Distribution of gene weights, with the top six genes with largest (absolute) weight highlighted.
(c) Distribution of factor values per batch of embryos, with cells coloured by the expression of the genes with highest weight.
(d) Gene set enrichment analysis applied to the Factor 3 weights (Methods).

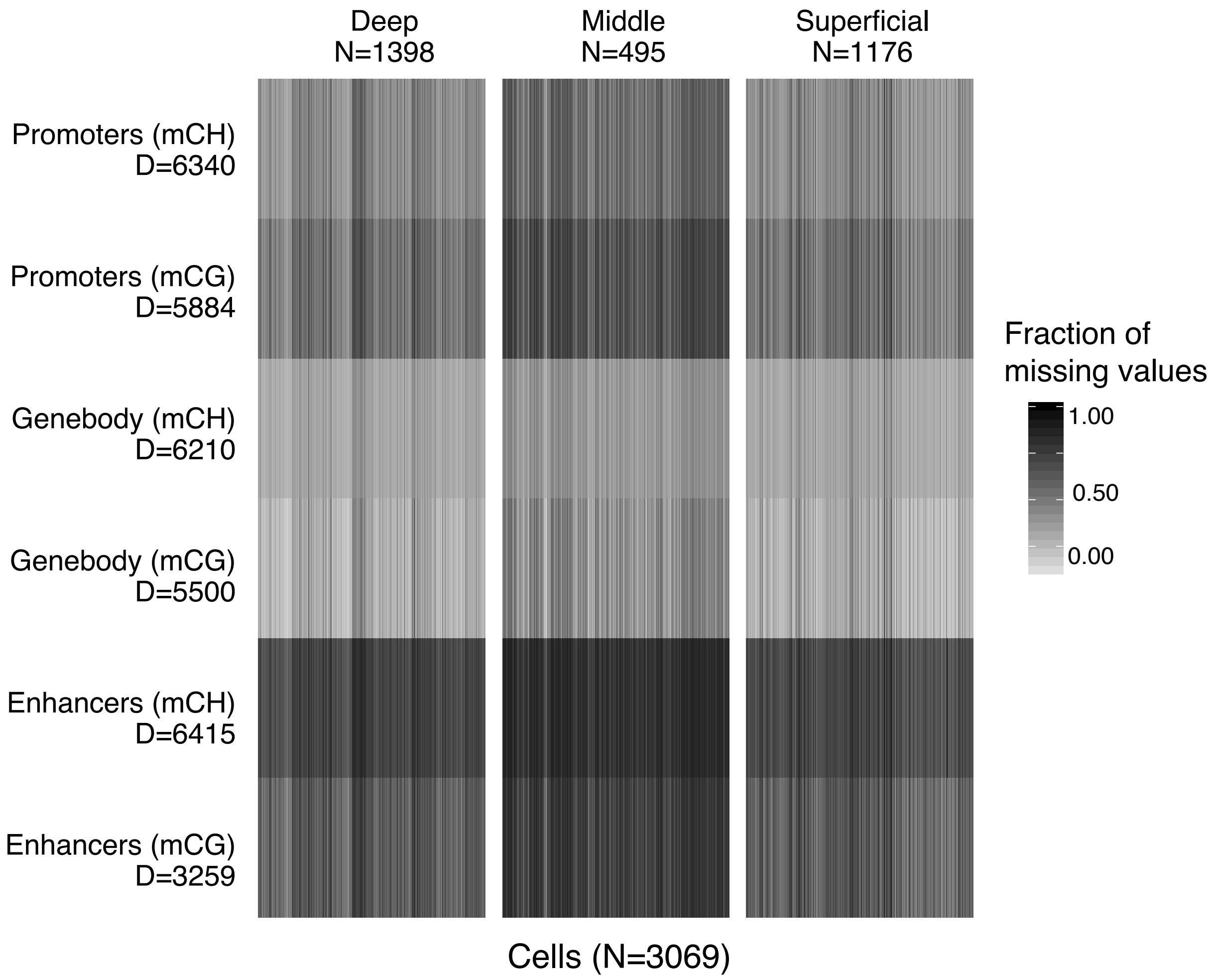

Fig. S7: Overview of the single-cell DNA methylation data set.
The tile plot shows the structure of the input data in terms of views (rows) versus groups (columns), with associated dimensionalities (D for features, N for samples). The color displays the fraction of missing values for each combination of sample and view.

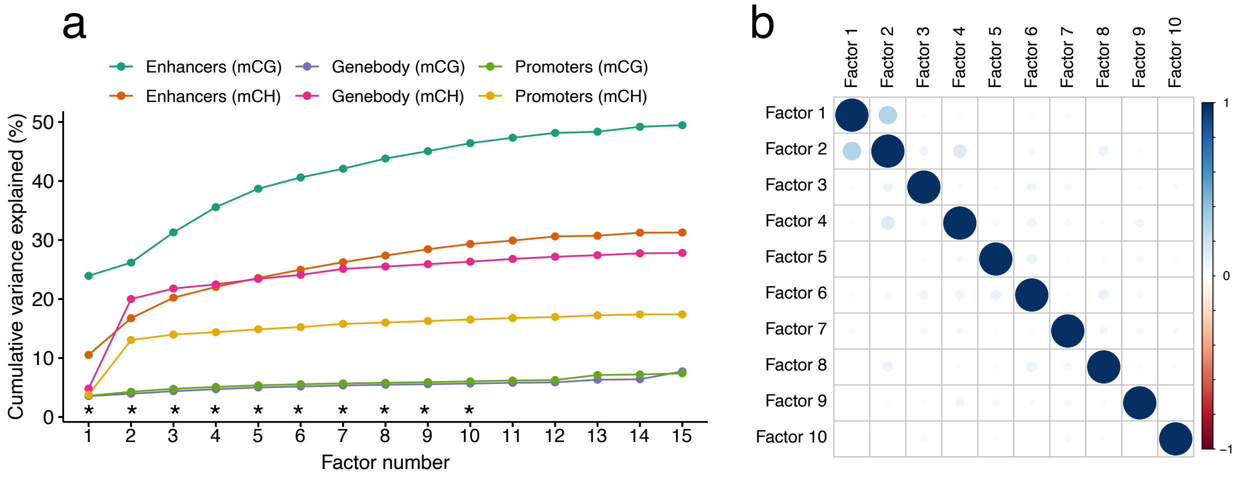

Fig. S8: Unsupervised characterisation of MOFA+ factors from the single-cell DNA methylation data set.

(a) Cumulative variance explained (per group, y-axis) versus factor number (x-axis). Asterisks indicate the factors that are selected for downstream analysis (minimum of 1% variance explained in at least one data modality).

(b) Pearson correlation coefficients between selected factors. In MOFA+ there are no orthogonality constraints, but the factors are expected to be largely uncorrelated.

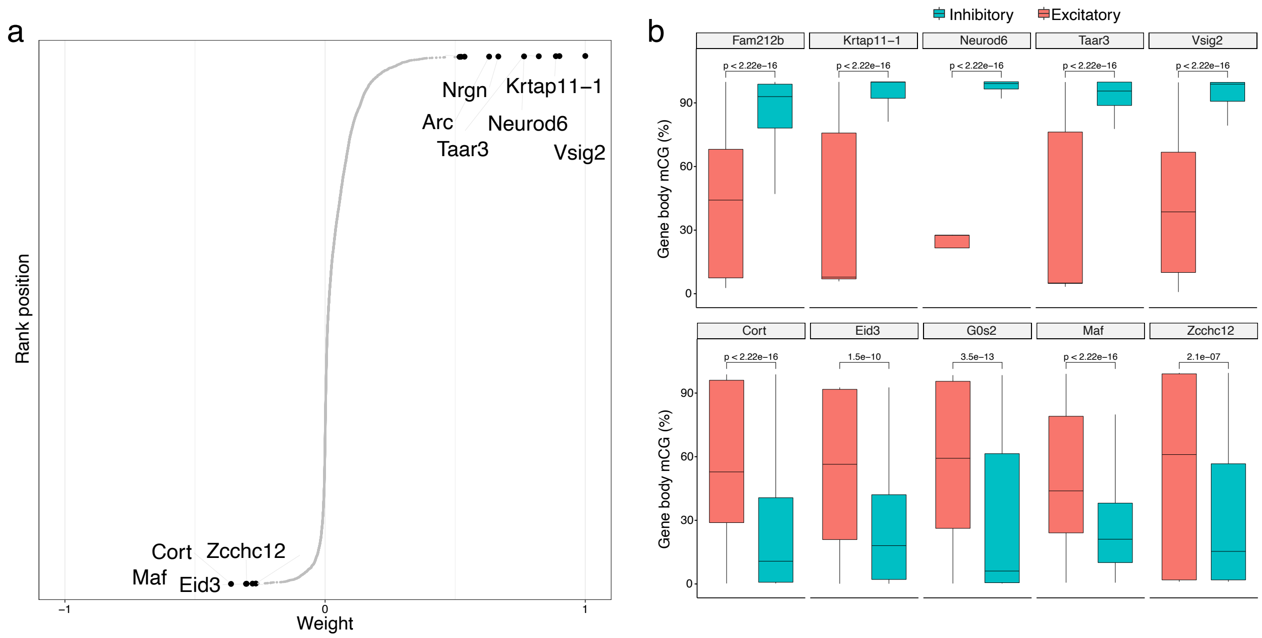

Fig. S9: Inspection of gene body mCG weights for Factor 1.

(a) Cumulative distribution of gene body mCG weights (x-axis) for Factor 1. Each dot corresponds to one gene, sorted by rank (y-axis) The weights provide a measure of feature importance, hence the higher the weight in absolute value, the higher the association between the feature and the factor (in this case, excitatory vs inhibitory neurons). The sign of the weight indicates the direction of the variability; positive weights indicate higher mCG in cells with positive Factor 1 values (inhibitory cells, see Figure 3b), whereas negative weights indicate lower mCG in cells with negative Factor 1 values (excitatory cells).

(b) Box plots comparing the distribution of gene body mCG levels (%) between excitatory and inhibitory neurons for the top 5 genes with the highest positive (top) or negative (bottom) weight. For each gene, a nominal p-value is reported using a t-test.

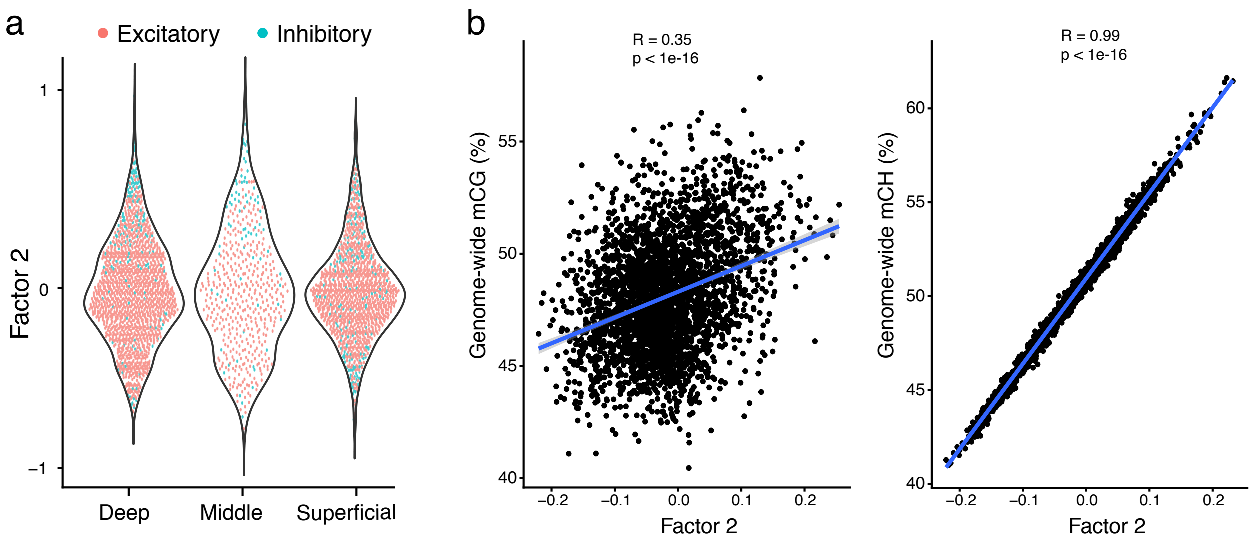

Fig. S10: Characterisation of Factor 2 as differences in global mCH levels.
(a) Beeswarm plots show the distribution of Factor 2 values for each cortical layer. Cells are coloured by neuron class.
(b) Correlation of Factor 2 values (x-axis) with global mCG levels (%, left) and global mCH levels (%, right). The blue line shows the linear regression fit.

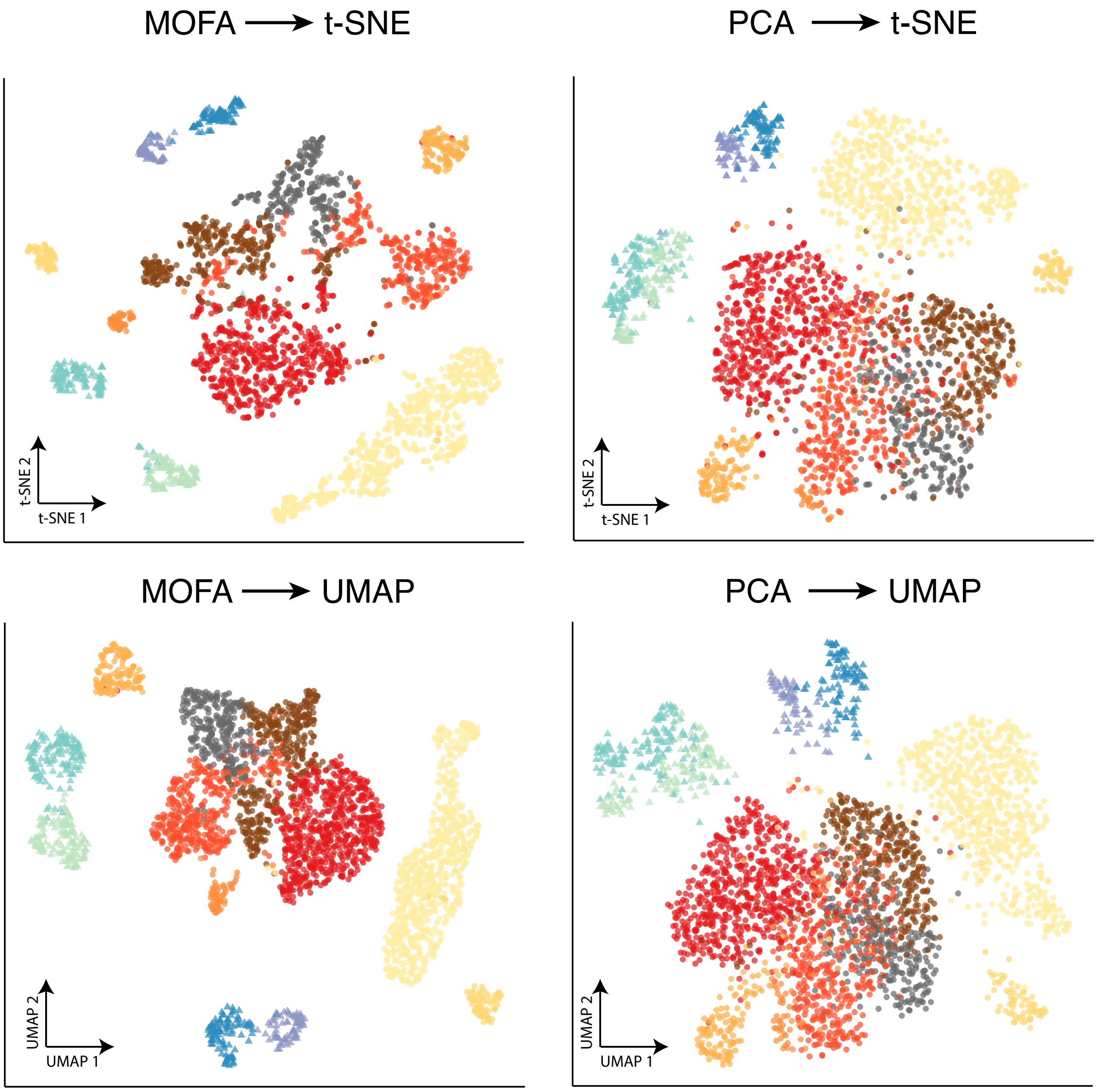

Fig. S11: Benchmarking MOFA+ factors and Principal Components as input to non-linear dimensionality reduction methods for DNA methylation data.
Plots display UMAP (bottom) or t-SNE (top) projections when using as input MOFA+ factors (left) or principal components (right). Each dot represents a cell, coloured by cell type assignments. Conventional implementations of Principal Component Analysis (*irlba* R package) do not handle missing values; missing values are thus imputed using feature-wise means.
To ensure a fair comparison we used the same number of PCs and MOFA+ Factors ($K = 15$).

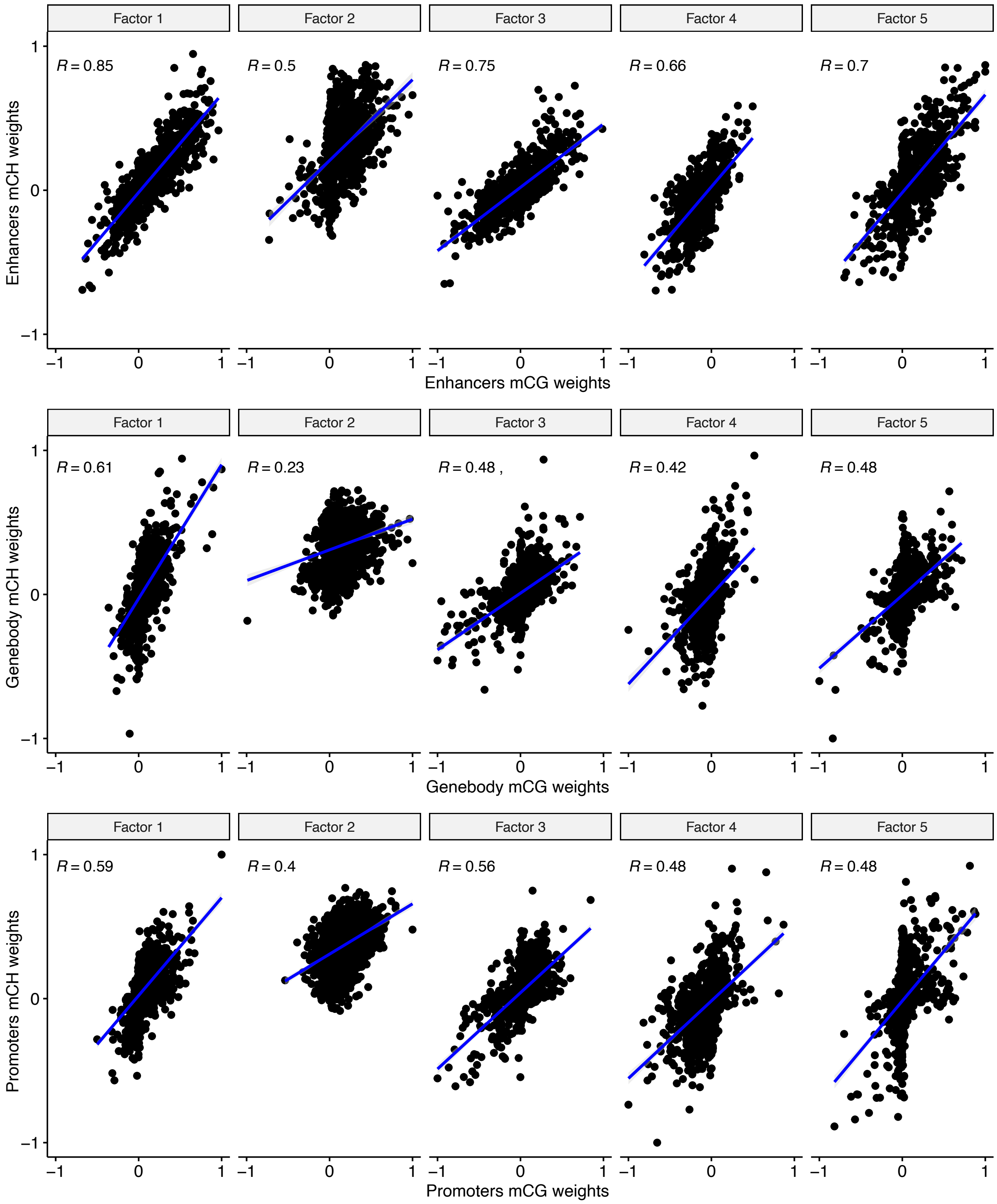

Fig. S12: mCH signatures are redundant to mCG signatures across multiple genomic contexts.

Scatter plots show the correlation between mCG weights (x-axis) and mCH weights (y-axis) for all combinations of factors (columns) and genomic contexts (rows). The blue lines display linear regression fits (all p-values$< 10^{-16}$). For each case we observe a significant positive correlation, indicating that the two DNA methylation signatures are not independent.

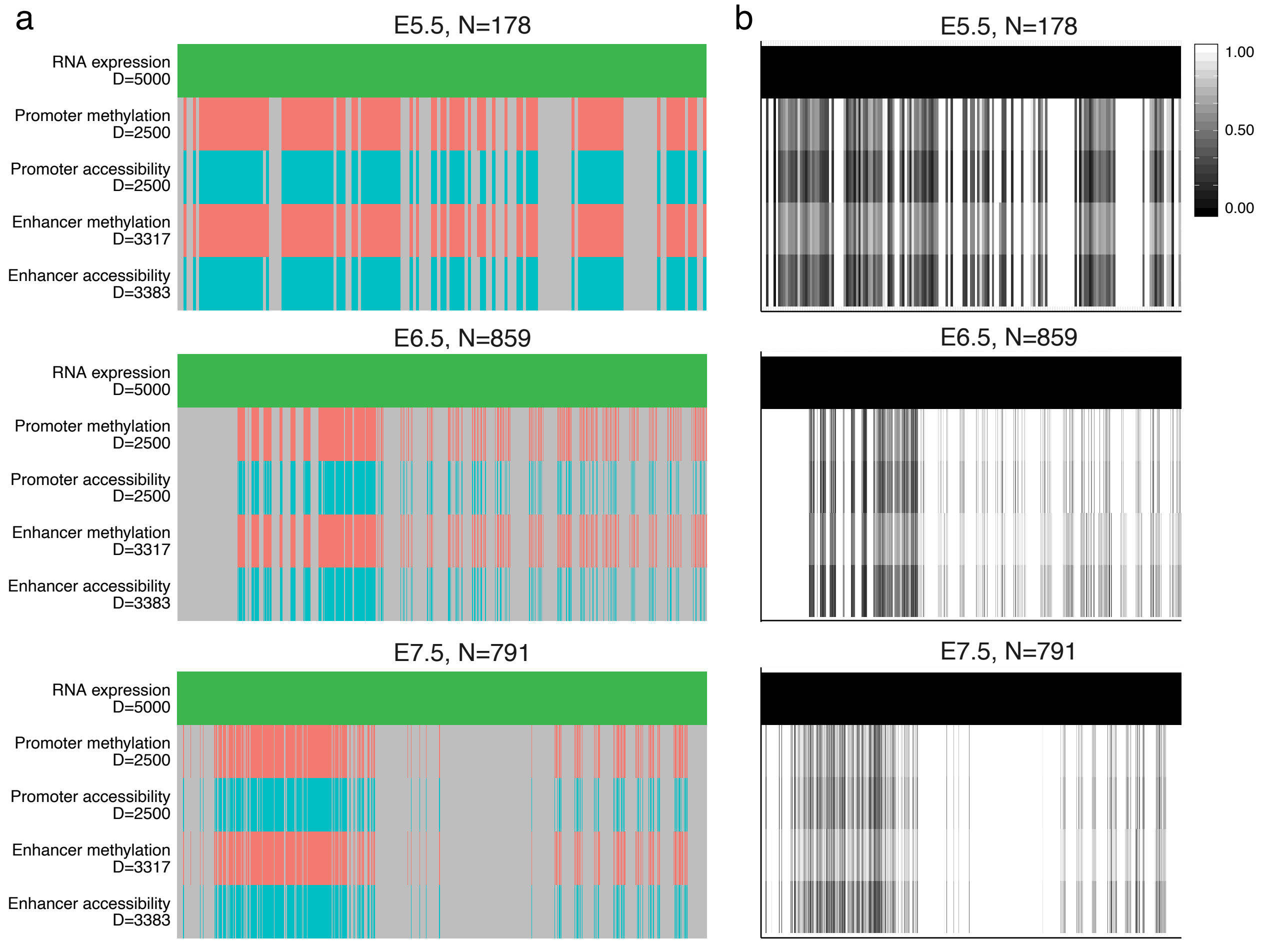

Fig. S13: Overview of multi-omic atlas of mouse gastrulation generated using scNMT-seq. (a) Structure of the input data in terms of views (x-axis) versus samples (y-axis). Each panel corresponds to a different group (embryonic stage). Grey bars represent missing views.
(b) Structure of the missing values in the data. For each cell and view, the colour displays the fraction of missing values.

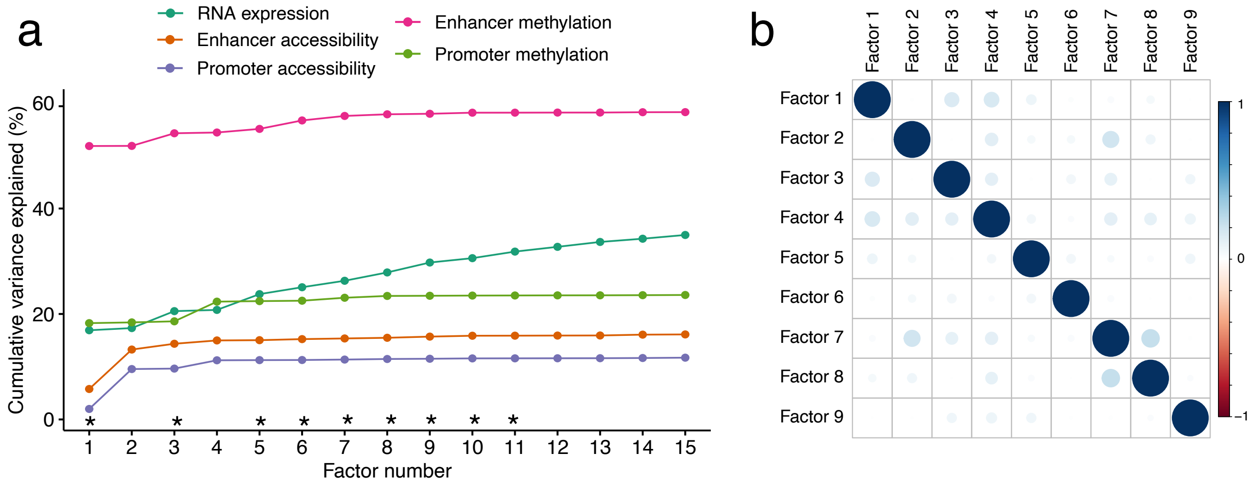

Fig. S14: Unsupervised characterisation of MOFA+ factors from the scNMT-seq gastrulation data set.
(a) Cumulative variance explained (per view, y-axis) versus factor number (x-axis). Asterisks indicate the factors that are selected for downstream analysis (minimum of 1% variance explained in the RNA expression).
(b) Pearson correlation coefficients between selected factors. In MOFA+ there are no orthogonality constraints, but the factors are expected to be largely uncorrelated.

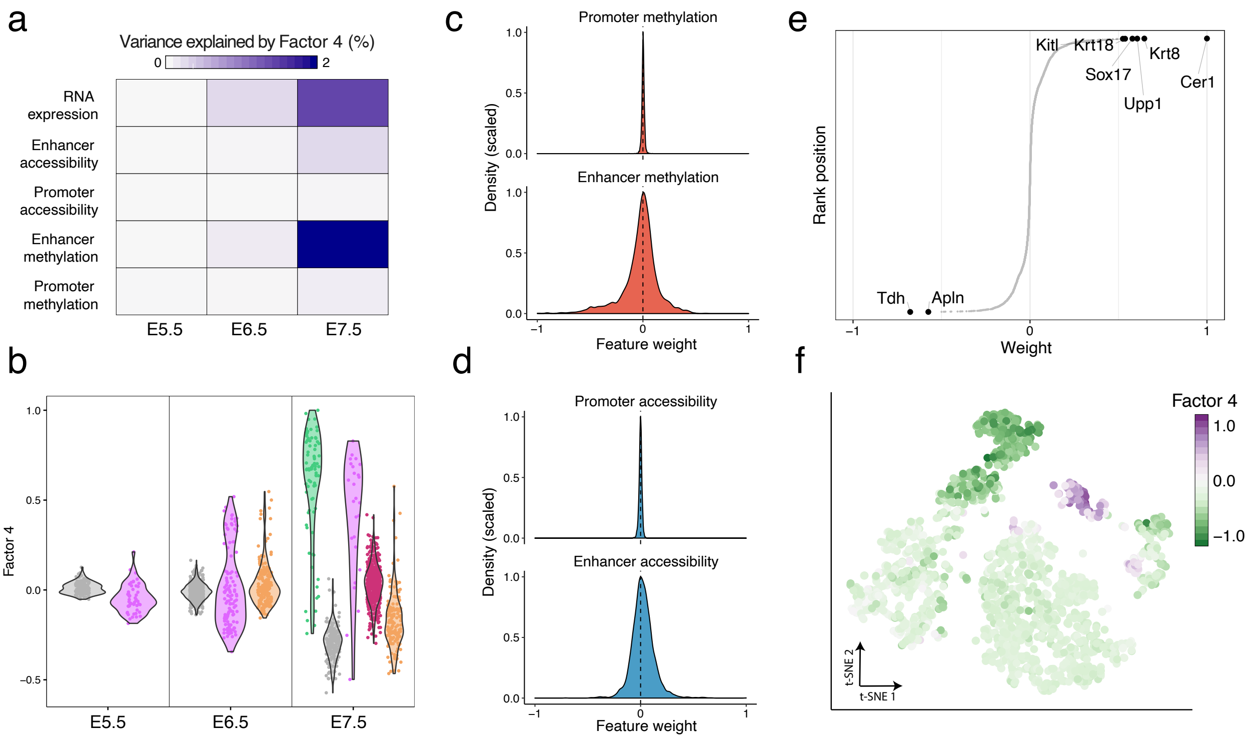

Fig. S15: Characterisation of Factor 4 as Embryonic endoderm commitment.
(a) Variance explained by Factor 4 in each group (embryonic stage, as columns) and view (rows).
(b) Distribution of Factor 4 values per group (embryonic stage, x-axis), with cells coloured by cell type assignment.
(c-d) Histograms display the distribution of (c) DNA methylation and (d) chromatin accessibility weights for promoters and enhancer elements.
(e) Distribution of RNA weights. The top genes with the highest (absolute) weight are labeled.
(f) Dimensionality reduction using t-SNE on the inferred MOFA factors. Cells are coloured by Factor 4 values.

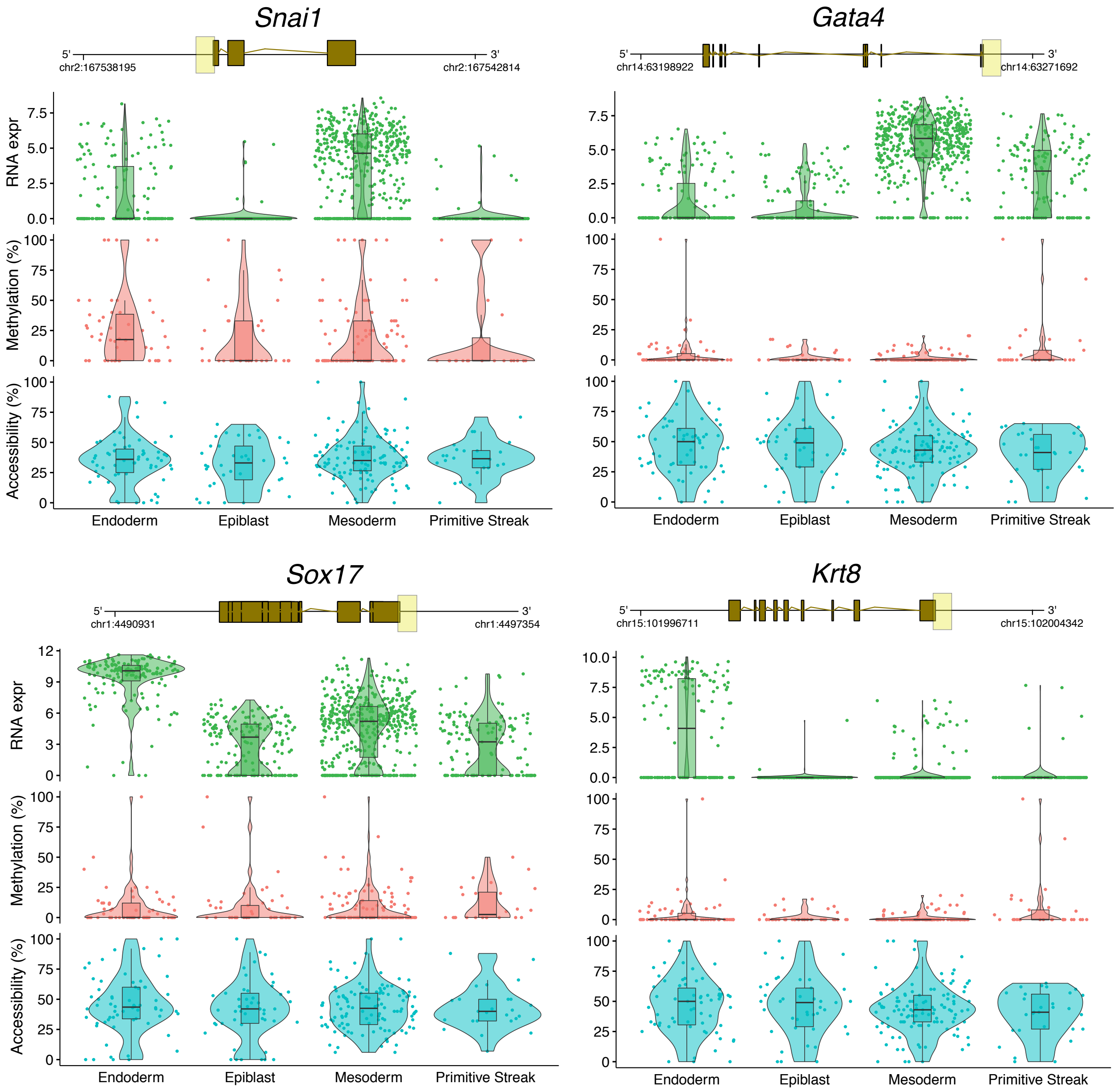

Fig. S16: Promoters of genes that display significant differential RNA expression between germ layers do not show differential epigenetic dynamics.
Box and violin plots show the distribution of RNA expression (log2 counts, green), DNA methylation (%, red) and chromatin accessibility (%, blue) levels per germ layer at E7.5. Each dot corresponds to a single cell. For each gene a genomic track is shown on the top. The promoter region that is used to quantify DNA methylation and chromatin accessibility levels (2kb upstream and downstream of the TSS) is highlighted in yellow.

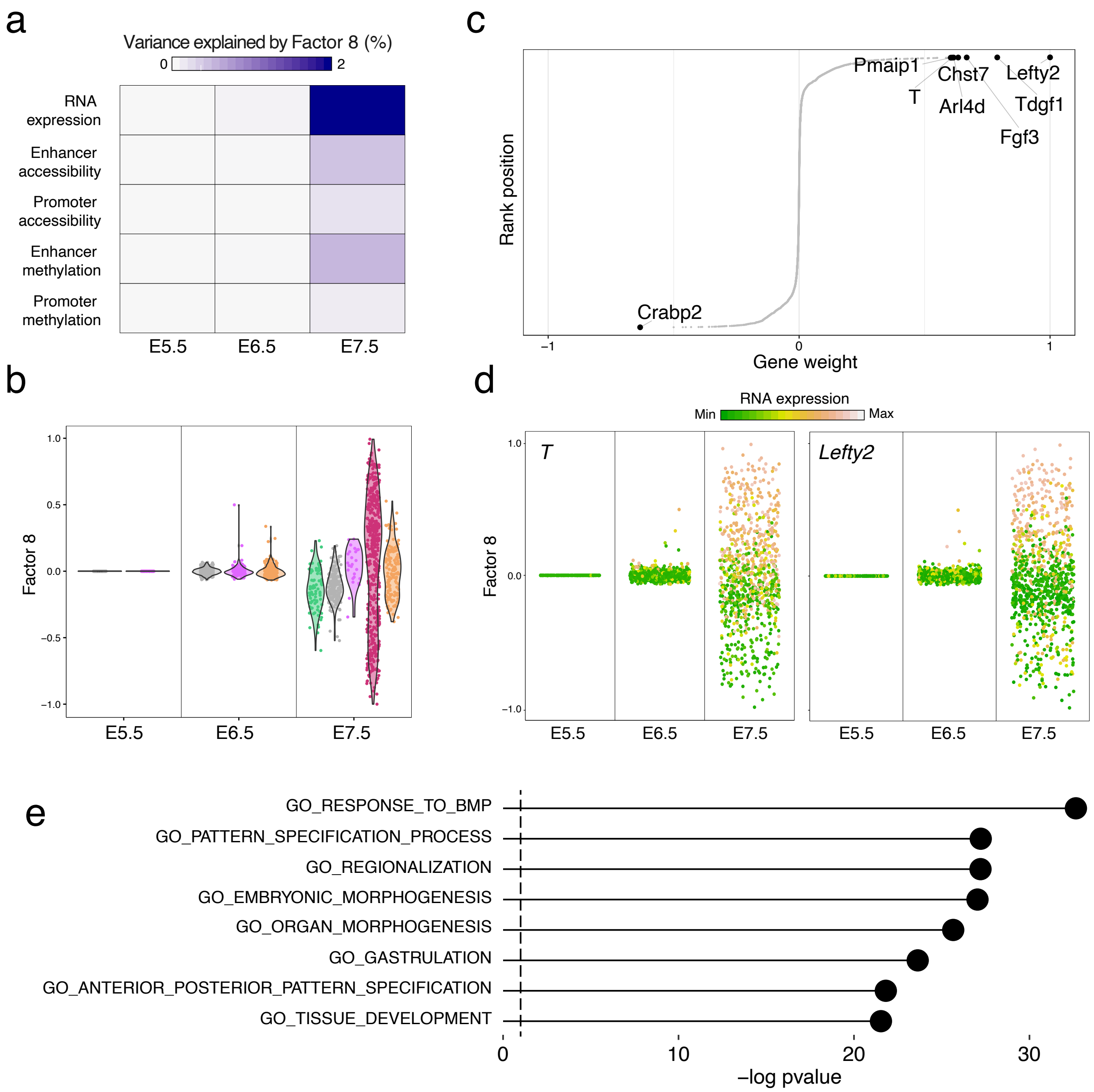

Fig. S17: Characterisation of Factor 8 as anterior-posterior axis formation in response to signalling cues.
(a) Variance explained by Factor 8 in each group (embryonic stage, columns) and view (rows).
(b) Distribution of Factor 8 values per group (embryonic stage, x-axis) with cells coloured by cell type.
(c) Distribution of RNA weights for Factor 8. The top genes with the highest (absolute) weight are labeled.
(d) Distribution of Factor 8 values per group (embryonic stage, x-axis), with cells coloured by the expression of T (left) and Lefty2 (right).
(e) Gene set enrichment analysis applied to the Factor 8 weights.

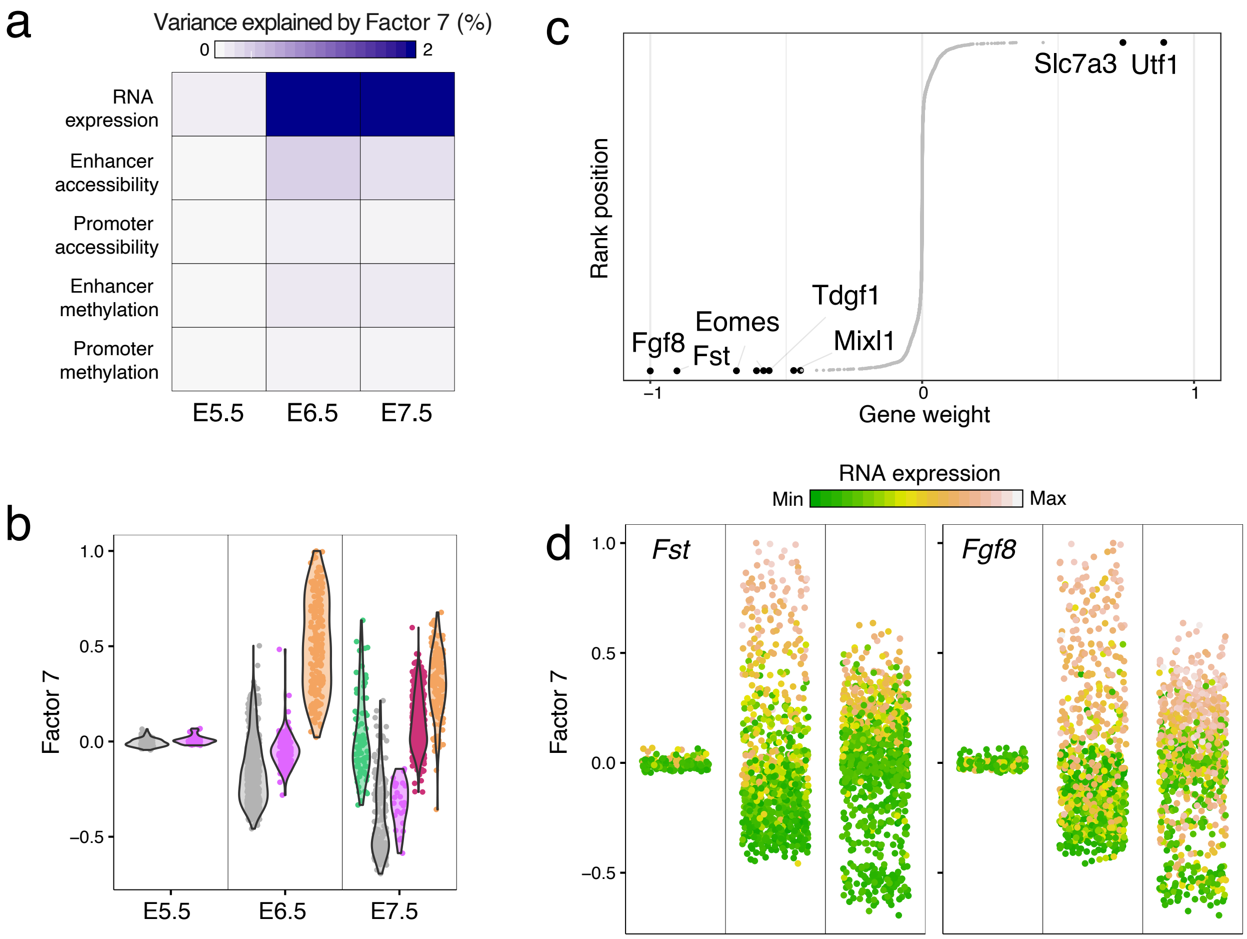

Fig. S18: Characterisation of Factor 7 as Primitive Streak formation.
(a) Variance explained by Factor 7 in each group (embryonic stage, columns) and view (rows).
(b) Distribution of Factor 7 values per group (embryonic stage, x-axis), with cells coloured by cell type.
(c) Distribution of RNA weights for Factor 7. The top genes with the highest (absolute) weight are labeled.
(d) Distribution of Factor 7 values per stage, with cells coloured by the expression of *Fst* (left) and *Fgf8* (right)

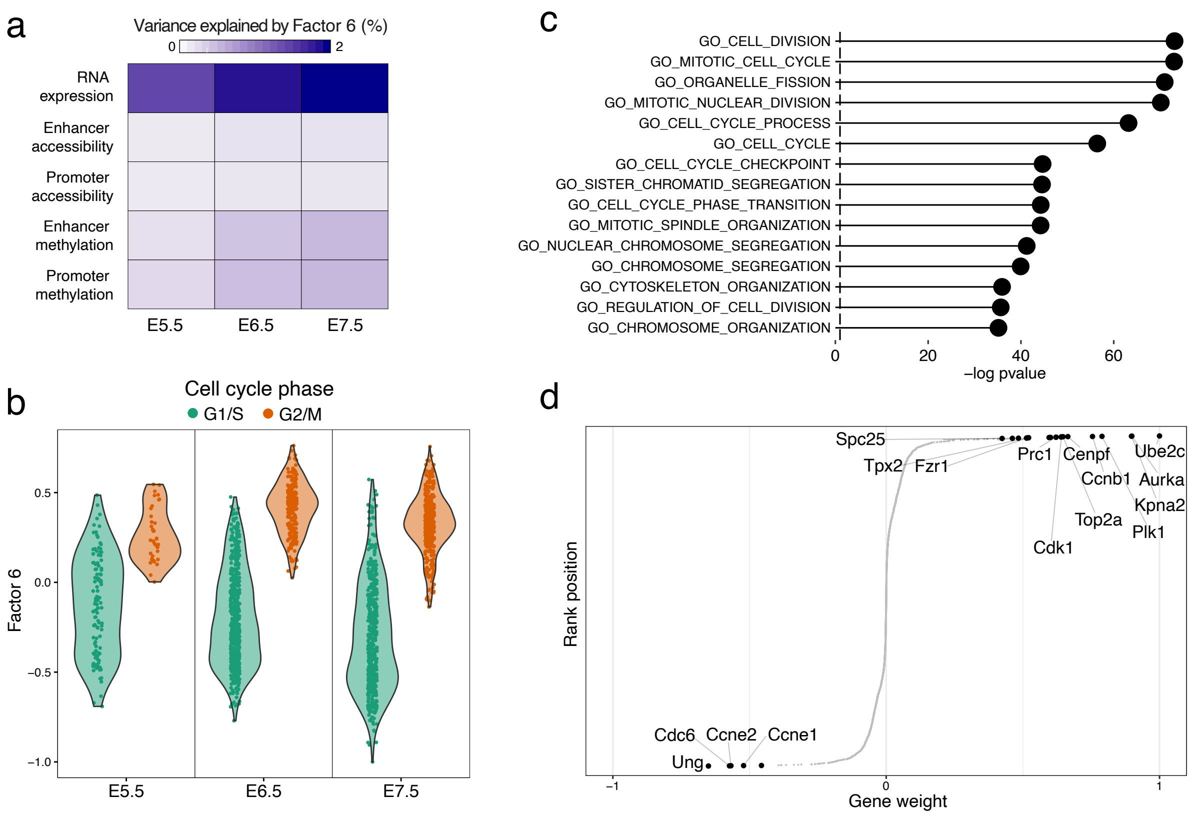

Fig. S19: Characterisation of Factor 6 as Cell Cycle.
(a) Variance explained by Factor 6 in each group (embryonic stage, columns) and view (rows).
(b) Distribution of Factor 6 values per group (embryonic stage, x-axis), with cells coloured by the inferred cell cycle state using *cyclone*.
(c) Gene set enrichment analysis applied to the Factor 6 weights.
(d) Cumulative distribution of RNA weights for Factor 6. The top genes with the highest (absolute) weight are labeled.

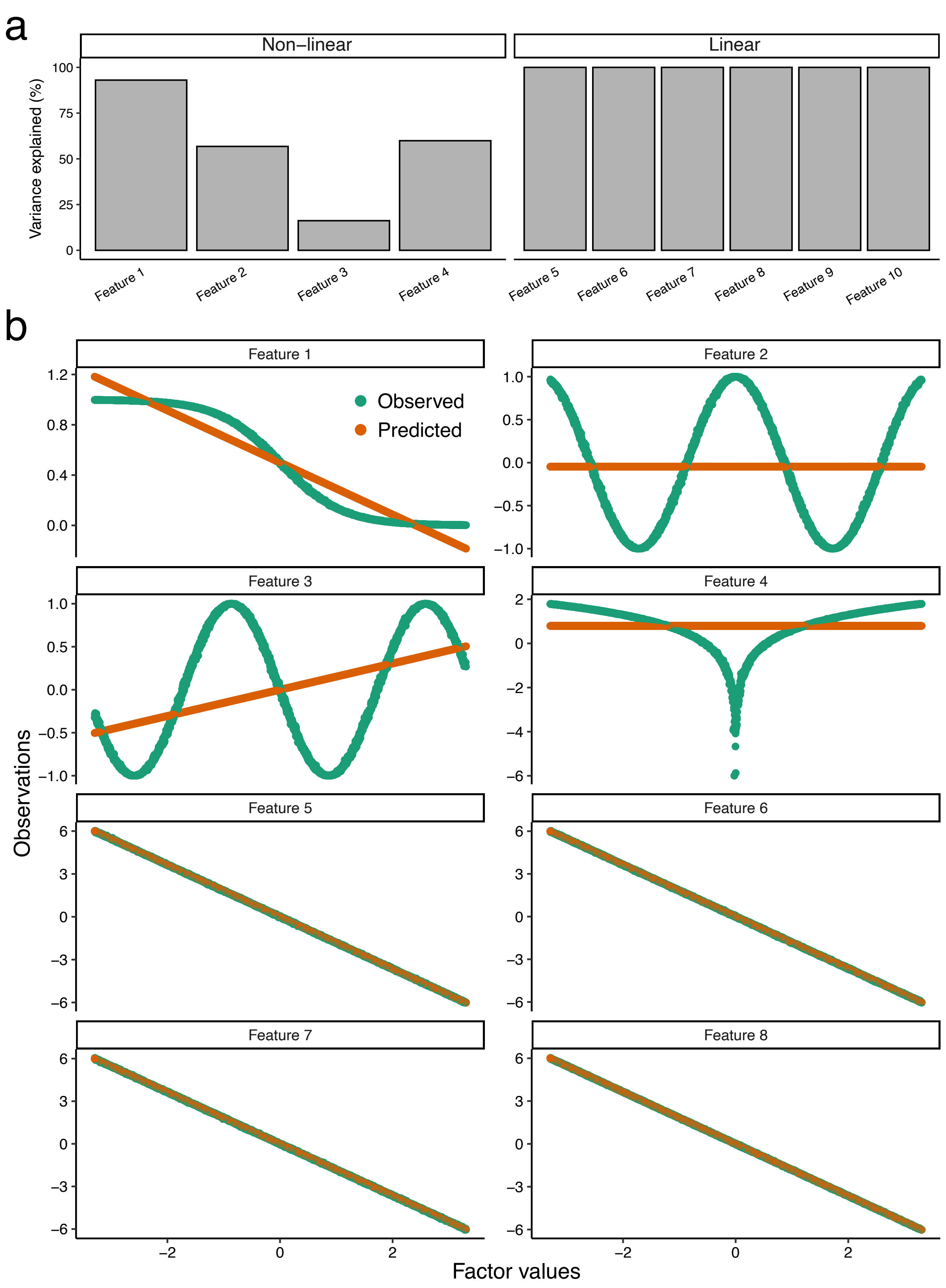

Fig. S20: MOFA has limited ability to detect strong non-linear relationships.
Data is simulated from the (linear) generative model with the following dimensionalities: $M = 1$ views, $G = 1$ groups, $D = 8$ features, $N = 1,000$ samples and $K = 1$ factors. Data was simulated without noise, but for the first four features we introduced different classes of non-linearities: sigmoid(x), cos(x), sin(x) and log(abs(x)) functions, respectively.
(a) Percentage of variance explained per feature.
(b) Predicted (orange) and observed (green) measurements versus Factor values.

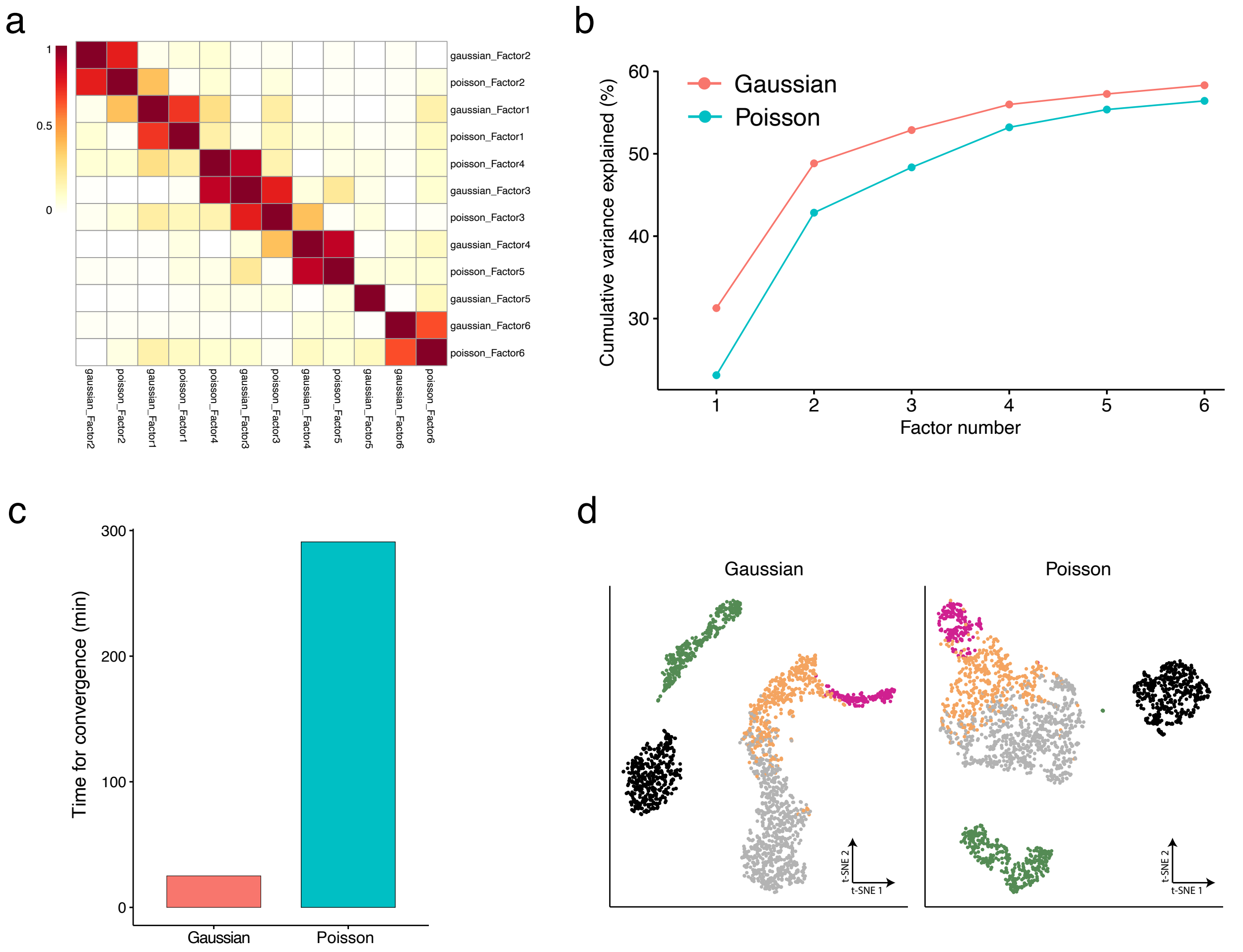

Fig. S21: Comparison of Poisson and Gaussian likelihood models in MOFA+ when applied to the scRNA-seq gastrulation data set.
(a) Heatmap shows Pearson correlation coefficient of Factor values between the two models. The blocks in the diagonal indicate that the factors are robustly found in the two model instances (except Factor 5 from the Gaussian likelihood, which is not recovered in the Poisson model).
(b) Line plots show the cumulative variance explained (per model, y-axis) versus factor number (x-axis). The gaussian model explains slightly higher variance than the Poisson model.
(c) Bar plots show the time for convergence (in minutes).
(d) Dimensionality reduction using t-SNE on the $K = 6$ inferred factors. Cells are coloured by cell type.
