# Supplementary material for: MOFA+: a statistical framework for comprehensive integration of multi-modal single-cell data
Source: Genome Biol. 2020 May 11;21:111. doi: 10.1186/s13059-020-02015-1 (PMC7212577; doi:10.1186/s13059-020-02015-1)
Supplement: Supplementary file 3 — Additional file 3. Supplementary Table 1, theoretical comparison with previous methods. [file 13059_2020_2015_MOESM3_ESM.pdf]

Publication	Scales to $> 1e5$ cells?	Multi-view	Multi-group	Missing values	Likelihoods
slalom [1]	No	No	No	No	ZI gaussian
pCMF [2]	Yes	No	No	No	ZI poisson
GFA [3]	No	Yes	No	Yes	Gaussian
ZIFA [4]	No	No	No	No	ZI gaussian
scVI [5]	Yes	No	Yes	No	ZI negative binomial
MSFA [6]	No	No	Yes	No	Gaussian
SDA [7]	No	No	Yes	No	Gaussian
ZINB-WaVE [8]	No	No	No	No	ZI negative binomial
AJIVE [9]	No	Yes	No	No	NA
DIABLO [10]	Yes	Yes	No	*	NA
scHPF [11]	Yes	No	No	Yes	Negative binomial
MOFA [12]	No	Yes	No	Yes	Gaussian/Poisson/Bernoulli
MOFA+	Yes	Yes	Yes	Yes	Gaussian/Poisson/Bernoulli

Table 1: **Overview of Factor analysis methods.**

*DIABLO handles missing data at random, but not samples missing an entire view.

References

[1] F. Buettner et al. "f-scLVM: scalable and versatile factor analysis for single-cell RNA-seq". In: *Genome biology* 18.1 (2017), p. 212.

[2] G. Durif et al. "Probabilistic count matrix factorization for single cell expression data analysis". In: *Bioinformatics* 35.20 (2019), pp. 4011–4019.

[3] A. Klami et al. "Group factor analysis". In: *IEEE transactions on neural networks and learning systems* 26.9 (2014), pp. 2136–2147.

[4] E. Pierson and C. Yau. "ZIFA: Dimensionality reduction for zero-inflated single-cell gene expression analysis". In: *Genome biology* 16.1 (2015), p. 241.

[5] R. Lopez et al. "Deep generative modeling for single-cell transcriptomics". In: *Nature methods* 15.12 (2018), pp. 1053–1058.

[6] R. De Vito et al. "Multi-study factor analysis". In: *Biometrics* 75.1 (2019), pp. 337–346.

[7] V. Hore et al. "Tensor decomposition for multiple-tissue gene expression experiments". In: *Nature genetics* 48.9 (2016), p. 1094.

[8] D. Risso et al. "A general and flexible method for signal extraction from single-cell RNA-seq data". In: *Nature communications* 9.1 (2018), pp. 1–17.

[9] Q. Feng et al. "Angle-based joint and individual variation explained". In: *Journal of multivariate analysis* 166 (2018), pp. 241–265.

[10] A. Singh et al. "DIABLO: from multi-omics assays to biomarker discovery, an integrative approach". In: *bioRxiv* (2018), p. 067611.

[11] H. M. Levitin et al. "De novo gene signature identification from single-cell RNA-seq with hierarchical Poisson factorization". In: *Molecular systems biology* 15.2 (2019).

[12] R. Argelaguet et al. "Multi-Omics Factor Analysis—a framework for unsupervised integration of multi-omics data sets". In: *Molecular systems biology* 14.6 (2018).
