# Supplementary material for: MOFA+: a statistical framework for comprehensive integration of multi-modal single-cell data
Source: Genome Biol. 2020 May 11;21:111. doi: 10.1186/s13059-020-02015-1 (PMC7212577; doi:10.1186/s13059-020-02015-1)
Supplement: Supplementary file 4 — Additional file 4. Review history. [file 13059_2020_2015_MOESM4_ESM.docx]

Review History

**First round of review**

**Reviewer 1**

**Were you able to assess all statistics in the manuscript, including the appropriateness of statistical tests used?**

Yes: no further assessment is required

**Were you able to directly test the methods?**

No

**Comments to author:**

Argelaguet et al. present MOFA+, a probabilistic framework for the integration of "multi-view" single-cell data. The model is an extension of the already published MOFA model. Compared to MOFA, MOFA+ offers several advantages, mostly (but not only) related to the scalability of the estimation procedure: it uses GPU-accelerated stochastic variational inference, and enforces sparsity both at the group and view level.

The manuscript presents a statistically sound method for the integration of multi-modal data. The authors convincingly present several use cases that uncover interesting biology, both using single-omic and multi-omic datasets. The method is a very timely contribution as large multi-omic studies at the single cell resolution are likely to become ubiquitous in the next few years.

I have a few comments and questions for the authors to address.

1. The three use cases presented in the manuscript are clearly in depth analyses of very interesting experimental datasets. However, it is not clear how the authors have chosen the number of factors and how they select the factors on which to focus on. In particular, the authors select a number of factors, but only focus on fewer when discussing the results. Is the order of the factors meaningful in this model? In general, more practical guidance on how to select the number of factors and how to interpret their values would be useful for the practitioners.

2. I understand that the original model was described somewhere else and I appreciate the extensive supplementary materials, but it would be nice to expand more on the assumptions of the model in the main text. In particular, the same k factors explain an underlying signal that is assumed common for each view. What are the consequence of this assumption? What if there are specific features that only occur in one view or in one group? Would a more flexible model with a common set of factors and specific sets be preferable? If not, why?

3. It is surprising that even though several likelihoods are implemented in MOFA+, a Gaussian likelihood is used for all views in all use cases, as far as I can tell. Wouldn't it be more beneficial to treat binary data with binomial likelihoods and counts with Poisson likelihoods? How did the authors transform the original measurements to obtain continuous variables? In general, it would be useful to provide practical guidance on which likelihood to use for each view (e.g., negative binomial for RNA-seq count data, binomial for SNPs, etc.).

4. In the embryogenesis case study (Figure 4) the authors state that "MOFA+ identifies 8 robust factors with a minimum variance explained of 1% in the gene expression view". Can the authors elaborate more on this? Does it mean that the gene expression variance explained by the model is 1%? Or does it mean that the 8th factor explains 1%? If the latter, what is the total variance explained for each view by the model? Ideally, this figure should be reported for all case studies. Interpreting factors that explain only 1% of the variance may be risky, so some practical guidance on interpretation may be good on this front as well.

5. Are the computational advantages reported in the manuscript only dependent on the use of the GPU or would the model be scalable even if run just on CPUs? It would be interesting to add one bar in Figure S2 relative to the performance of SVI on CPU to evaluate the relative contribution of the algorithm and the hardware.

Minor issues:

- Page 2, line 23: a citation to the original MOFA publication seems needed here.

- Page 5, line 18: I'm not sure that references 34,35 are appropriate here.

- Figure 2: how did the author get cell type information? Was this done through the MOFA+ analysis or based on prior information?

- Figure 3: similarly, how did the author obtain the deep/middle/superficial group? What about excitatory vs inhibitory? Are these a priori information or data driven? Also, labels in the UMAP in panel d need to be explained.

**Reviewer 2**

**Were you able to assess all statistics in the manuscript, including the appropriateness of statistical tests used?**

**Y**es: The paper described a novel method that is likely to have high impact

**Were you able to directly test the methods?**

No

**Comments to author:**

This is an well-articulated, well-written, timely article describing a method that is will be an important contribution to the mulit'omics of single cell data. Thank you for providing MOFA+ code in python, R, open source on github.

Please consider the below suggestions,

1) Please include a short section with recommendations for normalization, filtering and QC of data before MOFA+ (as this will be of interest to readers).

2) You state, each data type should include the same groups and cells. In a case where poor quality data was obtained on some cells in some data types, can data be imputed/excluded before MOFA+. How robust is the bayesian methodology to poor quality data or within data set batch effects?

3) Please provide short guidelines on selection of groups. Is it completely subjective? Are their limitations to the number of cells (or samples) per group. Could groups be defined by predicted cell lineage (cell types) with an experiment? Or should it be based on experimental design? If one selects groups that have a lot of within group noise (eg within group batch effect etc), are there simple fast tests of within/between group variance etc, to assess robustness of groups. Or should one use cross validation, training/test, or prediction strength as applied by Meng et al.,2019 in their paper on multi 'omics integration of bulk data using gene sets.

4) Will data types with larger number of features contribution greater variance to the results, if so, can please provide recommendations or approaches for 1) checking the contribution of each data type and 2) weighting the data types in the analysis. For example is order, feature size etc important. Is there a critical threshold at which the multi-omics is dominated by one data type?

5) please provide guidance on selection of the appropriate number of factors.

6) Whilst you apply different batch size/learning rate/forgetting rate parameters on simulated data in the supplement, the trade-off/impact of these are not discussed in detail. You state "their optimisation is likely to be important in some contexts". Which contexts? Which parameters are more critical multi'omics data integration. (Maybe provide detail in website of MOFA and provide link in text).

7) In data processing of the methylation data, data were transformed to M values and modeled with Gaussian likelihood. How critical is methylation data normalization/transformation. Does logit transformation impact MOFA+

8) In Figure 2b, most of the factor 1 values are negative. This is reminiscent of an arch effect in PCA, where the first component will have values skewed on the same side (positive or negative, most frequently negative). Can one observe horseshoes or arch-shaped effect in MOFA? (see tip 8, from Ten quick tips for effective dimensionality reduction. Nguyen & Holmes 2019, PLoS Comput Biol. 15(6):e1006907. , https://doi.org/10.1371/journal.pcbi.1006907)

**Authors Response**

**Point-by-point responses to the reviewers’ comments:**

We thank the reviewers for their positive critique of our work and for their constructive suggestions.

Reviewer #1

Argelaguet et al. present MOFA+, a probabilistic framework for the integration of "multi-view" single-cell data. The model is an extension of the already published MOFA model. Compared to MOFA, MOFA+ offers several advantages, mostly (but not only) related to the scalability of the estimation procedure: it uses GPU-accelerated stochastic variational inference, and enforces sparsity both at the group and view level. The manuscript presents a statistically sound method for the integration of multi-modal data. The authors convincingly present several use cases that uncover interesting biology, both using single-omic and multi-omic datasets. The method is a very timely contribution as large multi-omic studies at the single cell resolution are likely to become ubiquitous in the next few years.

I have a few comments and questions for the authors to address.

1. The three use cases presented in the manuscript are clearly in depth analyses of very interesting experimental datasets. However, it is not clear how the authors have chosen the number of factors and how they select the factors on which to focus on. In particular, the authors select a number of factors, but only focus on fewer when discussing the results. Is the order of the factors meaningful in this model? In general, more practical guidance on how to select the number of factors and how to interpret their values would be useful for the practitioners.

*Determining the most appropriate number of factors is in general an open question for factor analysis models. The optimal number of factors depends on the aim of the analysis, the dimensions of the assays, the complexity of the data, etc. If the aim is identifying the major sources of biological variation (as we consider in this manuscript) one typically considers the top factors (sorted by variance explained). In other tasks, such as imputation of missing values, even small sources of variation can be important and hence models should be trained with a large number of factors.*

*In practice, MOFA can automatically learn the number of active factors during training, but the user needs to specify (1) an initial number of factors, and (2) a minimum variance explained threshold per factor. As discussed, we cannot provide a straightforward answer for selecting a universally applicable threshold for the fraction of variance explained, as this will depend on the aim of the analysis. Nevertheless, as suggested by the referee, we have added two sections in Methods to guide the user how to determine the number of factors, and how factors should be interpreted (reproduced below):*

*“Determination of the number of factors*

*The selection of the number of factors is an important parameter of the training procedure. In MOFA+ we have implemented Automatic Relevance Determination priors (see Appendix Section 1.4) to prune unused factors during training. In practice, the user has to define the starting number of factors, and during model inference factors that do not explain any variation will be removed from the model. After the model is trained, the user can apply a second filtering by discarding factors that explain less than a pre-specified value of variance (in each data modality).*

*Interpretation of the factors*

*The MOFA factors capture the global sources of variability in the data. Mathematically, each factor ordinates cells along a one-dimensional axis centered at zero. Samples with different signs manifest opposite phenotypes along the inferred axis of variation, with higher absolute value indicating a stronger effect. Note that the interpretation of factors is analogous to the interpretation of the principal components in PCA.”*

*As mentioned above, in the examples described in the manuscript we focused on factors with the clearest biological interpretation (i.e. the top factors by explained variance), which provide a good illustration of the utility of the MOFA+ analysis pipeline. We did not include description of other factors for the sake of clarity, simplicity and space limitations. However, we now provide an improved characterisation of these additional factors in the associated	MOFA+	software	vignette (https://github.com/bioFAM/MOFA2/tree/master/MOFA2/vignettes)*

2. I understand that the original model was described somewhere else and I appreciate the extensive supplementary materials, but it would be nice to expand more on the assumptions of the model in the main text.

*As requested we have included additional information about MOFA and the underlying assumptions in the main text (lines 72-83).*

In particular, the same k factors explain an underlying signal that is assumed common for each view. What are the consequence of this assumption? What if there are specific features that only occur in one view or in one group? Would a more flexible model with a common set of factors and specific sets be preferable?

If not, why?

*As pointed out by the reviewer, the applicability of MOFA would be severely limited if it could only detect variation that is shared across all views. In fact, one of the major features of MOFA is its ability to distinguish signals that are shared across views from the signals that are unique to a single (or a subset) of views. Mathematically, this is achieved by the use of an Automatic Relevance Determination (ARD) prior in the weights of individual factors, which have view-specific hierarchical priors (see Appendix section 1.4).*

*We agree with the reviewer that this was not sufficiently clear in the main text, and we have now clarified this (lines 78-83).*

*This property is well illustrated in Figure S3 (reproduced below), where we simulate factors that have varying levels of variance explained across the different views and groups. For example, Factor 6 explains ~9% of variance in View 1 but 0% of the variance in View 0. Whereas standard Bayesian Factor Analysis (without ARD priors) struggles to disentangle these patterns of variation, MOFA+ is able to accurately recover the true simulated factor activity structure.*

*Figure S3. Validation of group-wise ARD prior in the factors using simulated data.*

*Data is simulated from the MOFA+ generative model with the following parameters: M=2 views, G=2 groups, D=1,000 features, N=1,000 samples and K=10 factors. Shown is the variance explained for every factor in each group (x-axis) and view (top row for view 0 and bottom row for view 1). We incorporate structure in the simulation process by turning some factors off in random sets of views and groups (ground truth shown on the right). Compared are MOFA+, MOFA v1 and Bayesian Factor Analysis (no sparsity priors).*

3. It is surprising that even though several likelihoods are implemented in MOFA+, a Gaussian likelihood is used for all views in all use cases, as far as I can tell. Wouldn't it be more beneficial to treat binary data with binomial likelihoods and counts with Poisson likelihoods? In general, it would be useful to provide practical guidance on which likelihood to use for each view (e.g., negative binomial for RNA-seq count data, binomial for SNPs, etc.).

*The reviewer is correct that we have used Gaussian likelihoods to model the different data modalities. We did this since, all of the settings considered in the manuscript, the measured readouts could be transformed into continuous measurements that are naturally modelled with a Gaussian distribution, as is commonly considered in normalisation of scRNA-seq (Lun et al. 2016), scNMT-seq (Clark et al. 2018) and single-cell DNA methylation data (Luo et al. 2017).*

*While in principle non-Gaussian likelihood models are indeed supported by MOFA and MOFA+, the use of efficient variational inference in conjunction with non-Gaussian likelihoods is statistically and computationally challenging (see discussion below). Given this, we suggest that these non-Gaussian likelihood models should only be used when required. Such cases include binary measurements (modelled with a Bernoulli likelihood) or low-count data (modelled with a Poisson likelihood).*

*From a mathematical perspective, variational inference in conjunction with non-Gaussian likelihoods, as we have considered it in our paper, is based the calculation of local variational bounds based on second-order Taylor expansions. This approach requires the introduction of additional approximations and variational parameters that must be inferred during inference, which significantly slows down the rate of convergence (see Section 4 of the Appendix of Argelaguet2018[2] for mathematical derivations). In addition, when applied in conjunction with Gaussian likelihood models, MOFA can learn a variance parameter for each feature (Tau in Equation 2 of the appendix), which means MOFA+ explicitly models heteroscedastic noise. When using non-Gaussian likelihoods, Tau has a fixed value and cannot be inferred from the model.*

*Having clarified this, we have tested the use of a Poisson likelihood to model the gastrulation scRNA-seq data set (only time point E7.0 for simplicity). The following figure shows a comparison of different statistics between a model trained with a Poisson likelihood and a model trained with a Gaussian likelihood. Overall, we observed that the solutions obtained from both models are qualitatively similar, however the Poisson model is significantly slower.*

*Figure: comparison of using a Poisson and Gaussian likelihood models in MOFA+ when applied to the scRNA-seq gastrulation data set*

*(a)	Heatmap shows Pearson correlation coefficient of Factor values between the two models. The blocks in the diagonal indicate that the factors are robustly found in the two model instances (except Factor 5 from the Gaussian likelihood, which is not recovered in the Poisson model)*

*(b)	Line plots show the cumulative variance explained (per model, y-axis) versus factor number (x-axis). The gaussian model explains slightly higher variance than the Poisson model.*

*(c)	Bar plots show the time for convergence (in min)*

*(d)	Dimensionality reduction using t-SNE on the 6 inferred factors. Cells are coloured by cell type.*

*In conclusion, if a data transformation can be safely applied to turn non-Gaussian readouts into a continuous nature (i.e., as is commonly applied to count data generated from sequencing assays), we recommend the use of Gaussian likelihoods.*

*As requested, we have clarified this and included practical guidelines for the use of non-Gaussian likelihoods in the Methods section and in the FAQ of the software (*[*https://github.com/bioFAM/MOFA2*](https://github.com/bioFAM/MOFA2)*).*

How did the authors transform the original measurements to obtain continuous variables?

*For single-cell RNA-seq data we used scran normalisation[3]. Briefly, the method takes as input the read counts, computes size-factors by pooling data across cells and applies a log transformation. Although some limitations exist with this approach (as recently discussed by Hafemeister2019[4] and Townes2019[5]), it nevertheless yields an accurate estimation of normalised RNA expression levels[6].*

*For single-cell DNA methylation (and single-cell NOME-seq data), we calculated DNA methylation (or chromatin accessibility) rates, which we subsequently transformed to M-values using a logit transformation[7] and modelled with a Gaussian likelihood. We adopted this transformation following the guidelines from [7], indicating that M-values have better statistical properties than the rate which is also strictly non-negative.*

*We have expanded the Methods section to describe in more detail the transformations used to process the data. In addition, we provide detailed guidelines and a FAQ section in the github repository (https://github.com/bioFAM/MOFA2) to ensure that users adopt appropriate data processing pipelines.*

4. In the embryogenesis case study (Figure 4) the authors state that "MOFA+ identifies 8 robust factors with a minimum variance explained of 1% in the gene expression view". Can the authors elaborate more on this? Does it mean that the gene expression variance explained by the model is 1%? Or does it mean that the 8th factor explains 1%?

*We apologise for the confusion - our statement means that each factor explains at least 1% of the total variance in the RNA expression. We have clarified this in the manuscript (line 232).*

*If the latter, what is the total variance explained for each view by the model? Ideally, this figure should be reported for all case studies.*

*As requested, we have added subfigures with the total variance explained estimates for each view in all case studies (Figure S4, S9 and S15). However, instead of using bar plots with the total variance explained, we have adopted a more informative visualisation that shows the cumulative variance explained (per group) versus factor number. An example is shown below for the 10x gastrulation data set (Figure 2).*

*Figure: cumulative variance explained (per group, y-axis) versus factor number (x-axis). Asterisks indicate the factors that are selected for downstream analysis (minimum of 1% variance explained across groups).*

Interpreting factors that explain only 1% of the variance may be risky, so some practical guidance on interpretation may be good on this front as well.

*We agree with the reviewer that interpreting factors that explain little variance is challenging, and this is the reason why we focus only on factors with variance explained values higher than a pre-specified amount.*

*Regarding the 1% variance threshold, in our experience, it is not uncommon for large single-cell data sets to recover biologically interpretable factors that explain relatively low amounts of variance. As the dataset size increases, even relatively small effects can be estimated with sufficient accuracy, and hence the 1% variance threshold may need to be adapted to the specific circumstance (tradeoff between assay noise, sample size and biological complexity).*

*An example is Factor 6, from the gastrulation scRNA-seq data set(Figure S6), which explains only 1.2 % of variance in the RNA expression but can be clearly associated with formation of mesenchymal cell types after E7.0.*

*A procedure that can be applied to evaluate the robustness of factors is to downsample the number of samples and/or the number of features and inspect how many factors are recovered. To demonstrate this, we downsampled the gastrulation scRNA-seq data set from 0% to 80% of the data set using a step size of 2.5% and we fit a MOFA+ for each condition with a fixed number of K=10 factors, and then we subset factors based on the 1% variance explained threshold. Subsequently, we calculated the Pearson correlation* *coefficient between the inferred factors across all models. As shown in the figure below, we find K=6 factors that are robustly found in downsampled data.*

*Figure: Factor robustness analysis in downsampled data. Heatmap shows the Pearson correlation coefficients between every pair of factors in the different model instances (one model per downsampling fraction). Each diagonal block represents a factor that is consistently learnt across all model instances.*

*Although this downsampling strategy can be useful to provide additional evidence about the importance of latent factors, there could be cases where the full data set is required to detect small yet important sources of variation. Hence, lack of robustness under downsampling does not necessarily imply that the factor is not biologically meaningful.*

5. Are the computational advantages reported in the manuscript only dependent on the use of the GPU or would the model be scalable even if run just on CPUs? It would be interesting to add one bar in Figure S2 relative to the performance of SVI on CPU to evaluate the relative contribution of the algorithm and the hardware.

*We thank the reviewer for raising this point which we did not explore in our first submission.*

*First, we want to point out that we have updated the convergence threshold in MOFA+. We were initially using a deltaELBO (the difference between the ELBO in iteration i and i-1) value of 1e-6. We noticed that this criterion was too strict, resulting in unnecessarily long training times. The new default value in the software, 1e-4, yields faster training times while effectively providing the near-identical solutions. This change is only important when comparing the scales of the y-axis in Figure S2 between the first submission and the revised manuscript - none of the other results change.*

*After this clarification, following the reviewer’s suggestion, we have updated Figure S2 to compare the convergence time estimates for SVI when using CPU versus GPU (reproduced below). We observe that for large sample sizes there is a speed improvement even when using CPUs, although these advantages become more prominent when using GPUs-based inference.*

*Figure S2. Evaluation of convergence speed for stochastic variational inference using simulated data.*

*Data is simulated from the generative model with varying sample sizes. The other data dimensions are fixed to M=3 views, G=3 groups, D=1,000 features (per view), and K=25 factors. Bar plots show the time elapsed for training MOFA+ models with stochastic variational inference (SVI). Colors represent different batch sizes (10%, 25% or 50%). The dashed line indicates the training time for standard variational inference (VI). VI models were trained using a single E5-2680v3 CPU. SVI models were trained either using a single E5-2680v3 CPU (first column) or using an Nvidia GTX 1080Ti GPU (second column).*

*Finally, we want to point out that although standard VI can also be accelerated with GPUs, the memory limitations of current GPUs (10 gigabytes for a 2018 Nvidia GTX 1080Ti used in this manuscript) renders SVI indispensable for large-scale (single-cell) datasets.*

*As requested, we have added clarifications about the stochastic inference scheme in the Methods, the FAQ section of the software (https://github.com/bioFAM/MOFA2) as well as in	the	SVI	vignette (https://github.com/bioFAM/MOFA2/blob/master/MOFA2/vignettes/stochastic_inference. Rmd).*

Minor issues:

-	Page 2, line 23: a citation to the original MOFA publication seems needed here.

*Done*

-	Page 5, line 18: I'm not sure that references 34,35 are appropriate here.

*Indeed, we have removed references 34 and 35 from the sentence.*

-	Figure 2: how did the author get cell type information? Was this done through the MOFA+ analysis or based on prior information?

*The cell types were annotated in Pijuan-Sala2019[8], which we downloaded from https://marionilab.cruk.cam.ac.uk/MouseGastrulation2018*

-	Figure 3: similarly, how did the author obtain the deep/middle/superficial group? What about excitatory vs inhibitory? Are these a priori information or data driven? Also, labels in the UMAP in panel d need to be explained.

*The cell metadata, including cortical layer and cell type information was extracted from Luo2017[9]. Briefly, to obtain the deep, middle and superficial neurons the cortical region was divided during dissection into three sections of approximately equal width such that “superficial layers” contained layers 1-3, “medium layers” contained layer 4, and “deep layers” contained layers 5-6 [Described in more detail in the supplementary information of Luo2017]. Hence, the cortical layer information was annotated before the actual sequencing. In contrast, the cell type annotations were derived from the sequencing data using an unsupervised clustering method called BackSPIN[10].*

Reviewer #2

This is an well-articulated, well-written, timely article describing a method that is will be an important contribution to the mulit'omics of single cell data. Thank you for providing MOFA+ code in python, R, open source on github.

Please consider the below suggestions,

1)	Please include a short section with recommendations for normalization, filtering and QC of data before MOFA+ (as this will be of interest to readers).

*As requested, we have added a section “Guidelines for data processing” in Methods where we provide recommendations for normalisation and feature selection. In addition, we have added an extensive FAQ section in the documentation of the package (https://github.com/bioFAM/MOFA2)*

2)	You state, each data type should include the same groups and cells. In a case where poor quality data was obtained on some cells in some data types, can data be imputed/excluded before MOFA+. How robust is the bayesian methodology to poor quality data or within data set batch effects?

*In general, probabilistic matrix factorisation methods are robust to poor quality (and sparse) data [11].*

*To demonstrate this, we simulated a single data modality resulting from K=10 factors by sampling from the generative model (as in Figures S1-S3) with the following likelihood:*

*To simulate different levels of noise we ranged the values of sigma from 0.1 (high signal-to-noise ratio) to 50 (low signal-to-noise ratio). Subsequently, we fit a MOFA+ model for each setting. The figure below shows that the variance explained by the model reduces as the noise levels increase (a), as expected. However, the inferred factors remain robust to increasing amounts of noise (b), owing to the model’s ability to simultaneously pool information from multiple features.*

(*a)	Variance explained (%) as a function of sigma (the variance of the normal distribution used to simulate the data). Each dot corresponds to a different model instance.*

*(b)	Heatmap showing the pearson correlation coefficients between every pair of factors in the different model instances (one model per value of sigma). Each diagonal block represents a factor that is consistently learnt across all sigma values. The figure indicates that all 10 factors are consistently found across all noise level settings.*

*We can also consider the most extreme case where all measurements in a group are driven by noise. To demonstrate this, we first simulate a structured data set with M=1 views (D=500 features) G=3 groups (N=1000 samples per group) and K=10 factors using the procedure described in the Methods section (see Figure S3). Fitting the MOFA+ model results in the following variance decomposition structure:*

*Figure: percentage of variance explained for each factor (y-axis) in each group (x-axis). A single view was used to simulate the data*

Next, we erase the signal in group “A” by randomly permuting the data, hence breaking any type of coordinated variability that could exist. This represents the (extreme) scenario where the measurements are only driven by "noise". Fitting the MOFA+ model results in the following variance decomposition structure, where any structure in group A is lost:

*Figure: percentage of variance explained for each factor (y-axis) in each group (x-axis). The input data was randomly permuted in group A. As expected, MOFA explains no variance in Group A but results for other groups are not affected*

*Finally, the reviewer also mentions batch effects, which is a specific source of variability that is driven by technical artifacts. In the case of an intra-group batch effect, we expect MOFA+ to capture a very strong factor that is exclusive to this group. To illustrate this, we have simulated a batch effect in the data set above by introducing an additional latent factor, where the factor values are sampled from a Bernoulli distribution with rate = 0.5 (on average 50% of samples assigned to batch A and 50% to batch B), followed by the addition of some gaussian noise:*

*When fitting the MOFA+ model to this data set, we indeed observe the presence of a strong Factor that explains variance exclusively in this group (Factor 1). Nevertheless, importantly, the remaining factors are not affected by the batch effect:*

*(a)	percentage of variance explained for each factor (y-axis) in each group (x-axis). Notice the presence of Factor 1, which captures the simulated batch effect, exclusively in group A*

*(b)	Beeswarm plot of Factor 1 values, coloured by batch label.*

*As we describe in the Methods and in the software webpage, to avoid this solution we recommend removing technical variation before fitting, when possible.*

3)	Please provide short guidelines on selection of groups. Is it completely subjective? Could groups be defined by predicted cell lineage (cell types) with an experiment? Or should it be based on experimental design?

*The selection of groups is driven by the hypothesis of the user. As the reviewer points out, they are typically based on the experimental design, but on some occasions one may want to explore data-driven groups.*

*Consider for example the brain single-cell DNA methylation data set (Figure 2). In our application we grouped cells by experimental design (cortical layers), but after identifying the major cell types (excitatory vs inhibitory neurons) one could attempt to group cells by this neuron type. When doing so, the task of MOFA+ is to identify which sources of coordinated variability are shared or unique between the two neuron types. We proceed to show the first three Factors that result from this model (keeping the same views)*

*Figure: Percentage of variance explained for the top three factors across the different groups (neuron class, x-axis) and views (genomic context, y-axis).*

*●	Factor 1 captures cell-to-cell variability in genome-wide mCH levels, which is observed in the two cell types.*

*●	Factor 2 is exclusive to excitatory cells and mostly driven by mCG and mCH in enhancer elements. This Factor captures the existence of (known) cell type heterogeneity within the excitatory neurons.*

*●	Factor 3 is exclusive to inhibitory cells and mostly driven by mCG and mCH in enhancer elements. Similarly, this Factor captures the existence of (known) cell type heterogeneity within the inhibitory neurons.*

*Figure: Characterisation of Factor 1 as cell-to-cell heterogeneity due to genome-wide differences in mCH.*

*(a)	Beeswarm plot of Factor 1 values, where cells are coloured by their global mCH values.*

*(b)	Plot of Factor 1 weights on the gene body mCH view,, where each dot corresponds to one gene. We observe that the distribution is shifted towards positive values, indicating that most gene bodies are affected in a similar fashion by this source of variation.*

*Figure: Characterisation of Factor 2 and Factor 3 as within-group heterogeneity for excitatory and inhibitory neurons, respectively.*

*(a)	Beeswarm plot of Factor 2 values, where cells are coloured by their excitatory subclass*

*(b)	Beeswarm plot of Factor 3 values, where cells are coloured by their inhibitory subclass Note: cell type labels are extracted from [9]*

*As requested, we have added a section in the Methods better explaining how the multi-group inference works and providing guidelines for the selection of groups.*

If one selects groups that have a lot of within group noise (eg within group batch effect etc), are there simple fast tests of within/between group variance etc, to assess robustness of groups. Or should one use cross validation, training/test, or prediction strength as applied by Meng et al.,2019 in their paper on multi 'omics integration of bulk data using gene sets.

*Within the MOFA+ framework, there is not a natural test that can be performed to assess the robustness or validity of groups before fitting the model. However, once the model is fit, the “quality” of the group structure can be rapidly assessed by visualising the variance explained plot. In particular, there are several outcomes that can result from a poor selection of groups. Here we outline the most frequent ones:*

*(1)	Samples within a group display no coordinated variation: in this case, MOFA+ will report no factors for the group (i.e. zero variance explained), which implies that the heterogeneity within the group is “noise”. This corresponds to the example above where we permute the measurements. In practice, this can occur if the groups are too granular, with the extreme case being one group = one sample.*

*(2)	Samples within a group display batch effects: in this case MOFA+ will learn a very strong factor that simply captures technical variation, hence potentially masking biological variability. This corresponds to the example above where we artificially introduce a batch effect in a single group. The solution in this case would be to either regress out the batch effect a priori, or treat the two batches as different groups.*

*(3)	Selection of groups with no meaningful biological differences: most multi-group biological data display structured variability, hence we expected some factors to be shared between groups while some factors will be exclusive to a single group. If the MOFA+ output results in all sources of variation being unique to single groups or rather shared between all groups, it generally implies a poor definition of groups, or views.*

*Consider the following example: let’s simulate a structured data set using the same procedure as above (reviewer’s point 2). Fitting the MOFA+ model with the “correct” definition of groups results in a highly structured variance decomposition:*

*Figure: percentage of variance explained for each factor (y-axis) in each group (x-axis)*

*Next, we will permute the group assignment labels, which represents the case where the groups are poorly defined. Fitting the MOFA+ model with the “incorrect” definition of groups results in an unstructured variance decomposition plot, where all factors are shared between all groups:*

*Figure: percentage of variance explained for each factor (y-axis) in each group (x-axis).*

*Note that all factors are shared between all groups and hence the variance decomposition pot is unstructured.*

*In conclusion, the definition of groups is hypothesis-driven, and typically motivated by the experimental design. There is no “right” or “wrong” definition of groups, but some definitions will be more useful than others. A quick approach to assess the validity of groups is to inspect the variance explained plot as illustrated above. If the groups are too granular, the model will not recover significant amounts of variation. If a group contains (within-group) batch effects, the model will likely recover a strong factor that captures this variation, which should be easily identified by the user when plotting the factor values. Finally, if the groups are not “interesting”, this can result in an unstructured model output, with all factors being shared across all groups.*

*As the reviewer points out, there are other approaches to assess the robustness of groups, including cross-validation approaches but also downsampling and/or bootstrapping of samples within groups. We have added this in the FAQ section of the software (https://github.com/bioFAM/MOFA2, see points 1.4 and 1.5)*

Are their limitations to the number of cells (or samples) per group?

*Statistically speaking, there is no limitation in the number of cells per group, or in the number of groups. In practice, however, the reviewer is correct to point out that the number of cells (or samples) per group does have an impact on the output. As described above, if the number of cells allocated to a group is very small, the model will not recover significant amounts of variation. In general, the more samples / cells per group, the more complexity there will exist in the dataset, which can manifest itself in retrieval of a higher number of factors.*

*We can provide experiments based on simulated data (see below for the analogous question on the number of features per view), but it is impossible to provide specific thresholds for real data. The signal that can be extracted from small groups will depend on the data modality, the degree of structure within the dataset, the levels of noise and on how strong the sample imbalance is between groups.*

*To aid the application of MOFA in practice, we have added a section in the Methods providing guidelines for the selection of groups.*

4)	Will data types with larger number of features contribution greater variance to the results, if so, can please provide recommendations or approaches for 1) checking the contribution of each data type and 2) weighting the data types in the analysis. For example is order, feature size etc important. Is there a critical threshold at which the multi-omics is dominated by one data type?

*In general, it is true that the number of features can influence the contribution of a data type to the latent space. This is because the objective function (the evidence lower bound, ELBO) does not weight the different data modalities according to their number of features.*

*Having said this, we find the model to be very robust to differences in the dimensionality of the feature space. To illustrate this we simulated data with M=2 views and K=3 factors (one shared and two specific to each view) with an increasing imbalance in the number of features (from 1x (equal dimensions) to 500x as many features in the larger view).*

*Even for very large discrepancies in the number of features the model still learnt all three factors including the one factor specific to the smaller view and the inferred factors explained all variation generated by simulated factors (see figure below).*

*(a) Factors inferred using MOFA+ on simulated data with increasing imbalance between views. Data was simulated from 2 view-specific factors and one shared factors, with 200 features in the small view and 200*x features in the large view, varying x from 1 to 500. (b) Variance explained by the inferred factors in each view compared to the variance explained by the simulated factors (dashed line) for varying imbalance between views (x-axis)*

*However, on real data, all structure will rarely be captured by the factors and here indeed the model will be more prone to learn factors that explain variation in the larger view. As an illustration we simulated data from 2 views with a total of 9 factors (each a strong, a medium and a weak factor with activities in both views, the small or the large view) and constrained the model to learn 6 factors only. Here, with increasing imbalance the model will fail to learn the factors which are specific to the small view and focus instead on the (weaker) signal in the large view. Of note, however, the model will still be able to find the shared factors even if there is a large difference in the number of features.*

*Factors inferred using MOFA+ on simulated data with increasing imbalance between views. Data was simulated from 3 view-specific factors per view and 3 shared factors with varying strength (low, medium, strong), with 100 features in the small view and 100*x features in the large view.*

*In practice, a generic feature imbalance threshold at which this type of effect will occur is hard to define. As the simulation above illustrates, it will depend on the structure contained within each view and how strong the feature imbalance is. The simplest remedy that we suggest (in Methods and in the FAQ) is to subset highly variable features to avoid very strong imbalances.*

A second option, which the reviewer suggests, is a weighting scheme. We have implemented this in MOFA+ by weighting the contribution of each view on the ELBO (the objective function), as follows: Denoting all view-wise variables with Xm variables with X we use:

and remaining

where γm is pre-specified as:

*The plot below shows the results from the simulation in Fig. 2 when applying the weighting scheme. Notably, the model is now able to better capture the signal from the small view.*

*However, we want to note that this weighting scheme is a heuristic that is applied at the objective function; it is subjective, and it is not clear how to specify it in the Bayesian generative model. Hence, there are no theoretical guarantees about its behaviour. Consequently, we prefer not to use the weighting scheme as a default criteria but leave it as an option for the user in case of a strong feature imbalance.*

*Figure on simulated data with imbalance between views. Inference is performed using a weighted ELBO in the MOFA+ model.*

*To aid the application of MOFA in practice, we have added a section in the Methods providing guidelines for the selection of data modalities.*

5)	please provide guidance on selection of the appropriate number of factors.

*As requested also by the first reviewer (see Reviewer 1 point #1), we have included a section in Methods on how to determine an appropriate number of factors.*

6)	Whilst you apply different batch size/learning rate/forgetting rate parameters on simulated data in the supplement, the trade-off/impact of these are not discussed in detail. You state "their optimisation is likely to be important in some contexts". Which contexts? Which parameters are more critical multi'omics data integration. (Maybe provide detail in website of MOFA and provide link in text).

*We agree with the reviewer that a more in-detail description of the stochastic inference algorithm was missing. We have included a discussion on the trade-off of the hyperparameters in the Methods section. In addition, we have added a vignette (https://raw.githack.com/bioFAM/MOFA2/master/MOFA2/vignettes/stochastic_inference. html) with reproducible code and with practical guidelines on how and when to use it.*

*The sentence “their optimisation is likely to be important in some contexts” was indeed not precise and we have rephrased it. We found that the hyperparameters batch size = 50%, learning rate = 0.75 and forgetting rate = 0.25 provide stable and sensible results in a variety of simulated and real data sets, but we cannot rule out that they may have to be optimised in other data sets with different characteristics. Hence, as discussed in the corresponding	vignette*

*(https://raw.githack.com/bioFAM/MOFA2/master/MOFA2/vignettes/stochastic_inference. html) we advise the user to perform model selection with a grid-search approach.*

7)	In data processing of the methylation data, data were transformed to M values and modeled with Gaussian likelihood. How critical is methylation data normalization/transformation. Does logit transformation impact MOFA+?

*We transformed to M-values following the guidelines from[7], where they suggest that the M-value has better properties for downstream analysis of DNA methylation levels.*

*However, we acknowledge that the logit transformation has little impact on the MOFA+ output (at least for this data set). To demonstrate this, we fit the MOFA+ model for the Luo2017 data set [9] using the M-values and the beta-values using a fixed number of K=10 factors. For both cases, the factor values are highly consistent between the two models (Figure below, a) and their variance explained estimates are similar (Figure below, b)*

*Figure: comparison of Beta-values vs M-values for the single-cell DNA methylation application*

*(a)	Heatmap showing the Pearson correlation coefficients between every pair of factors in the two model instances. Each diagonal block represents a factor that is consistently learnt in the two models.*

*(b)	Cumulative variance explained (%, y-axis) versus factor number (x-axis). The two lines*

*represent either input Beta-values (red) or M-values (blue)*

8)	In Figure 2b, most of the factor 1 values are negative. This is reminiscent of an arch effect in PCA, where the first component will have values skewed on the same side (positive or negative, most frequently negative). Can one observe horseshoes or arch-shaped effect in MOFA? (see tip 8, from Ten quick tips for effective dimensionality reduction. Nguyen & Holmes 2019, PLoS Comput Biol. 15(6):e1006907. , <https://doi.org/10.1371/journal.pcbi.1006907>)

*Horseshoe or arch effects can indeed introduce artifacts in dimension reduction methods. These effects can also be observed in MOFA+ if a nonlinear pattern dominates the data.*

*To evaluate this for MOFA+ we adopted the reproducible rmarkdown file (https://www.huber.embl.de/users/whuber/pub/horseshoe.html) developed by Dr. Wolfgang Huber and simulated data from a non-linear kernel for each feature using the squared exponential kernel with varying length scales. As mentioned in the resource, this leads to patterns in the covariance matrix that are concentrated on the diagonal and have an exponential decay away from the diagonal. Learning a 2-dimensional embedding using MOFA+ results in a horseshoe or arch effect (see Figure below) with increasing bendiness for decreasing length scale. This artifact will be visible in any method based upon a linear kernel such as factor models or PCA, CCA-like methods, as a squared exponential kernel cannot be reproduced faithfully by a linear kernel (see also [12,13]). Like other linear dimensionality reduction methods, users of MOFA+ hence need to be aware of such artifacts when interpreting the results. We have added a note on this to the FAQ (in https://github.com/bioFAM/MOFA2).*

*This figure shows the results of MOFA factors learnt on simulated data with a non-linear kernel. A single view was simulated with each feature independently following a multivariate normal distribution with covariance function between samples given by a squared exponential kernel with varying length scales (A ls = 0.05, B ls =0.2, C ls=0.5). The left panels show the resulting covariance matrix between samples, the right panels the 2-dimensional latent space inferred by MOFA using 2 factors. A similar effect can be observed for other linear dimension reduction methods. See https://www.huber.embl.de/users/whuber/pub/horseshoe.html.*

*[1]	Seeger M. Fast Variational Bayesian Inference for Non-Conjugate Matrix Factorization Models n.d.*

*[2]	Argelaguet R, Velten B, Arnol D, Dietrich S, Zenz T, Marioni JC, et al. Multi-Omics Factor Analysis-a framework for unsupervised integration of multi-omics data sets. Mol Syst Biol 2018;14:e8124.*

*[3]	Lun ATL, McCarthy DJ, Marioni JC. A step-by-step workflow for low-level analysis of single-cell RNA-seq data with Bioconductor. F1000Res 2016;5:2122.*

*[4]	Hafemeister C, Satija R. Normalization and variance stabilization of single-cell RNA-seq data using regularized negative binomial regression. Genome Biol 2019;20:296*

*[5]	Townes FW, Hicks SC, Aryee MJ, Irizarry RA. Feature selection and dimension reduction for single-cell*

*[6]	RNA-Seq based on a multinomial model. Genome Biol 2019;20:295.*

*Vieth B, Parekh S, Ziegenhain C, Enard W, Hellmann I. A systematic evaluation of single cell RNA-seq*

*[7]	analysis pipelines. Nat Commun 2019;10:4667.Du P, Zhang X, Huang C-C, Jafari N, Kibbe WA, Hou L, et al. Comparison of Beta-value and M-valuemethods for quantifying methylation levels by microarray analysis. BMC Bioinformatics 2010;11:587.*

*[8]	Pijuan-Sala B, Griffiths JA, Guibentif C, Hiscock TW, Jawaid W, Calero-Nieto FJ, et al. A single-cell molecular map of mouse gastrulation and early organogenesis. Nature 2019;566:490–5.*

*[9]	Luo C, Keown CL, Kurihara L, Zhou J, He Y, Li J, et al. Single-cell methylomes identify neuronal subtypes and regulatory elements in mammalian cortex. Science 2017;357:600–4.*

*[10]	Zeisel A, Muñoz-Manchado AB, Codeluppi S, Lönnerberg P, La Manno G, Juréus A, et al. Brain structure. Cell types in the mouse cortex and hippocampus revealed by single-cell RNA-seq. Science 2015;347:1138–42.*

*[11]	Koren Y, Bell R, Volinsky C. Matrix Factorization Techniques for Recommender Systems. Computer 2009;42:30–7. https://doi.org/10.1109/mc.2009.263.*

*[12]	Brazill TJ, Grofman B. Factor analysis versus multi-dimensional scaling: binary choice roll-call voting and the US Supreme Court. Soc Networks 2002;24:201–29.*

*[13]	Diaconis P, Goel S, Holmes S. Horseshoes in multidimensional scaling and local kernel methods. Ann Appl Stat 2008;2:777–807.*

**Second round of review**

**Reviewer 1**

I would like to thank the authors for satisfactorily addressing all my questions in their response.

I only have a couple of minor suggestions that I trust the author will be able to implement before publications.

Overall, I want to congratulate the authors for a very good method and paper!

Minor suggestions:

- I may be biased, but I find very interesting the comparison of Gaussian models on transformed data vs. modeling using a likelihood that (in principle) better fit the data structure. For this reason, I really appreciated the time and effort put by the authors to address the related point in my first review and I particularly liked their Figure in response to my point #3, comparing Poisson and Gaussian models for RNA-seq. I believe that other readers would benefit from the insights in this figure and I suggest the authors to add it as a supplementary figure.

- I may have missed something, but one of the variables that can be used to define “groups” in MOFA+ is batch. However, in the guidelines for data processing it is suggested to “a priori” regress out batch effects. Isn’t this in contrast with using batch to define groups in MOFA+? Or is this the advice when there is a different definition of “group”? In general, batch effects are hard to deal with, since they may be confounded with donor, treatment, or other critical variables if the experimental design is not careful. It seems a bit too simplistic to advise to regress out batch effects as a general recommendation and I suggest that the authors change the language in their suggestion to reflect these concerns.
